# Distinct endothelial cells in chronic thromboembolic pulmonary hypertension

**DOI:** 10.1038/s44325-025-00072-8

**Published:** 2025-07-02

**Authors:** Hon-Sum Jeffrey Man, Yidan D. Zhao, Usman Asghar, Licun Wu, Jonathan C. Yeung, John Thomas Granton, Marc de Perrot

**Affiliations:** 1 Departments of Respirology and Critical Care Medicine, University Health Network and Sinai Health System, Divisions of Respirology and Interdepartmental Division of Critical Care, University of Toronto, Toronto, Canada; 2 Latner Thoracic Research Laboratories, Toronto General Hospital Research Institute, University of Toronto, Toronto, Canada; 3 Department of Immunology, University of Toronto, Toronto, Canada; 4 Division of Thoracic Surgery, University Health Network, University of Toronto, Toronto, Canada

**Keywords:** Angiogenesis, Blood flow, Thrombosis, Thromboembolism, Thrombosis, Cardiovascular diseases

## Abstract

Chronic thromboembolic pulmonary hypertension (CTEPH) is a life-threatening condition characterized by unresolved thrombi obstructing the pulmonary arteries. Endothelial cells (ECs) are essential regulators of thrombosis and thrombus resolution, yet their molecular phenotypes in CTEPH are incompletely understood. Using single-cell RNA sequencing of CTEPH surgical specimens and control ECs from the Human Lung Cell Atlas, we uncover marked EC heterogeneity in CTEPH. Compared to controls, CTEPH ECs display marked phenotypic shifts, with the loss and emergence of distinct EC subpopulations, including a unique subset co-expressing pulmonary and bronchial EC markers. Our analysis reveals perturbed endothelial thrombosis regulation, encompassing coagulation, fibrinolysis, inflammation, TGF-**β** signaling, and angiogenesis, while identifying novel target genes. These findings provide a comprehensive view of endothelial contributions to delayed thrombus resolution, offering new insights into the pathophysiology of CTEPH and potential therapeutic targets.

## Introduction

Chronic thromboembolic pulmonary hypertension (CTEPH) is a leading cause of pulmonary hypertension (PH) and results in progressive right heart failure and death^1^. CTEPH is generally considered to be a complication of pulmonary embolism^2^. In CTEPH, the nonresolving pulmonary embolism is characterized by intraluminal fibro-inflammatory tissue that obstructs the pulmonary arteries. The primary treatment of CTEPH involves lifelong anticoagulation and pulmonary endarterectomy (PEA), which removes the intraluminal material^3,4^. Other treatments for CTEPH include balloon pulmonary angioplasty (BPA) and targeted medical PH therapy^5^. New treatments are needed because up to 40% of patients have nonoperative management and up to 51% have persistent PH after surgery^5–9^.

However, the pathogenesis of CTEPH remains unclear. PEA can dramatically improve pulmonary pressures despite removing a limited amount of PEA tissue, suggesting that the fibro-inflammatory tissue plays a vital role in the severity of pulmonary vascular resistance^10^. Histologic examination of PEA tissue reveals inflammatory cell infiltration and fibrosis^11^. Therefore, understanding the pathologic cellular and molecular patterns that lead to the pathologic transformation of pulmonary artery thromboembolism is an essential step for identifying new targets for therapy in CTEPH^12,13^.

In most cases of pulmonary embolism, complete thrombus resolution occurs within 3 months^14^, and the incidence of CTEPH after pulmonary embolism ranges from 0.57–4.7% in published studies^15^. A major question in CTEPH pathogenesis is why pulmonary embolism does not resolve in this subset of patients. Blood-based coagulopathies are risk factors for the development of CTEPH, but a majority of patients do not have an identified blood-based coagulopathy^2,15,16^. Furthermore, 25% of patients with CTEPH have no history of symptomatic pulmonary embolism^8^, suggesting that local factors may contribute to the development of CTEPH. Indeed, increased expression of type 1 plasminogen activator inhibitor (PAI-1), the principal inhibitor of tissue- or urokinase-type plasminogen activator, is found within CTEPH surgical specimens but not in the systemic circulation^17,18^. Thus, systemic coagulopathy does not fully explain the development of CTEPH.

The classical triad (Virchow’s triad) of thrombosis proposes that the endothelium is a cornerstone of thrombosis along with blood flow and blood-based coagulation factors^19,20^. Normally, the endothelium provides an anticoagulant surface. In its role in regulating thrombosis, the endothelium can contribute to platelet reactivity, coagulation, fibrinolysis and vascular contractility^20^. Physiologically, endothelial cells contribute to vascular repair^21^ and thrombus resolution through recanalization of thrombus by angiogenesis^5,20,22–25^. On the other hand, altered endothelial phenotypes may contribute to CTEPH through aberrant angiogenic patterning, vascular inflammation and endothelial-mesenchymal transition^5,26–29^.

Heterogeneity of EC phenotypes within vascular beds is critical to vascular homeostasis and injury response^20,30–37^ and emerging single-cell RNA sequencing (scRNAseq) reveal that distinct EC subpopulations within the lung are vital for homeostasis^34,35,38^. Therefore, we hypothesized that altered EC subpopulations and phenotypes contribute to the pathology of CTEPH.

We report that the CTEPH PEA specimen has high cellular diversity with diverse immune cell types comparable to atherosclerosis, another chronic inflammatory vascular disorder. In keeping with bronchial collateralization to PEA specimens^11^, we find a mixed origin of ECs arising from both the pulmonary and bronchial circulation. This heterogeneity of origin contributes to some differences in EC phenotype in CTEPH.

By controlling for both EC cell types, we observe pronounced pathologic shifts in EC phenotypes. We find a unique subpopulation of CTEPH ECs that display a convergence of phenotypes with co-expression of both pulmonary and bronchial artery EC markers. Trajectory analysis is consistent with increased laminar shear stress from bronchial collateralization as one factor in shifting pulmonary artery EC phenotypes in CTEPH.

Our scRNAseq data provides a transcriptional profile of endothelial dysfunction from thrombogenesis through thrombus resolution with simultaneous abnormalities in endothelial regulation of coagulation, fibrinolysis, inflammation, TGF-**β** signaling and angiogenesis. Our data, controlled for endothelial subtypes, confirm perturbed gene expression of vWF^39^, TGF-**β**^25,40^, ANGPT2^41^. Furthermore, we identify additional endothelial target genes across all domains of thrombus evolution in CTEPH, including dysregulation of coagulation (TFPI), fibrinolysis (SERPINE1 encoding PAI-1), endothelial activation (SELP) and angiogenesis (DLL4, NOTCH1). We find that imbalance of angiogenic potential previously observed^5,23,27^ may be due, at least in part, to “tip/stalk” imbalance with decreased “tip cells” and increased “stalk cells”. Overall, these data provide new insights into the pathophysiology of delayed thrombus resolution in CTEPH.

## Results

### Single-cell RNA sequencing identifies 20 distinct cell populations in human PEA specimens

**Fig. 1:**
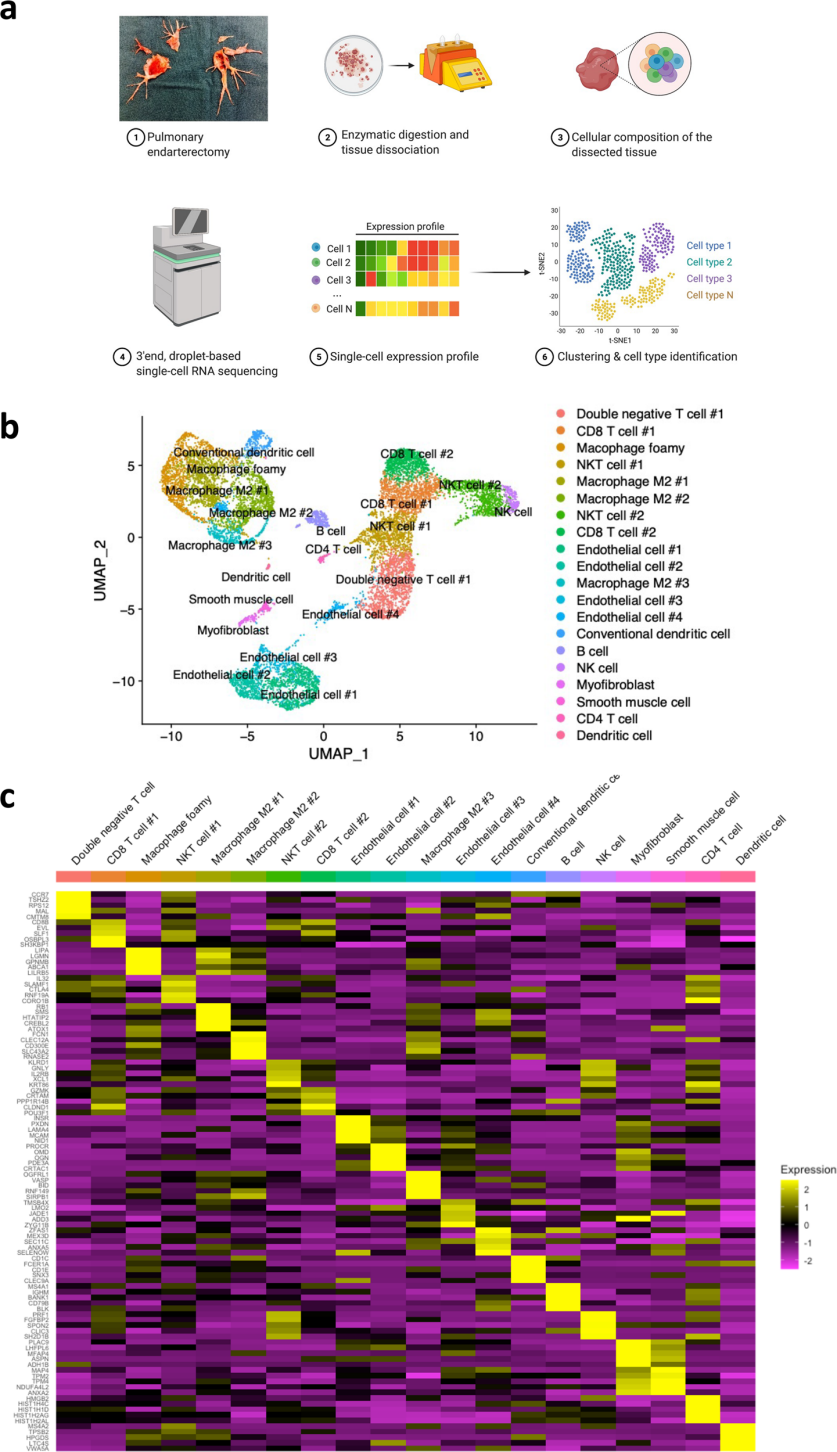
Single-cell RNA sequencing identifies 20 distinct cell populations in human pulmonary endarterectomy specimens. **a** Experimental setup: pulmonary endarterectomy specimen tissue was dissociated physically and digested enzymatically. Dissociated cells underwent 3’end, droplet-based single-cell RNA sequencing, generating single-cell expression profiles. Principal component analysis, clustering, differential gene expression, and universal manifold approximation and projection (UMAP) visualizations were performed. Figure created with BioRender.com. **b** UMAP visualization of clustering revealed 20 cell populations. Population identities were determined based on marker gene expression (Supplementary Table 2). **c** Heatmap of top 5 markers for each cell cluster illustrating a subset of marker gene expression patterns between clusters.

Five human PEA specimens (three male, two female) were used to prepare scRNAseq libraries (Table 1, Fig. 1a, Supplementary Table 1). After filtering cells by the number of reported genes and mitochondrial content, we obtained transcriptional profiles for 11068 cells clustered into 20 cell populations (Fig. 1b, c, Supplementary Fig. 1a–e). We manually assigned cell types to each cluster based on lineage marker expression (Supplementary Table 2). Almost all clusters were represented in each sample, with some variability in the distribution of clusters in each sample (Supplementary Fig. 2). We observed six non-immune clusters representing endothelial cells (ECs), smooth muscle cells and myofibroblasts and 14 immune clusters from myeloid and lymphoid lineage. T cells were the most abundant population, followed by macrophages.

**Table 1:** Patient characteristics for scRNAseq

Number	Gender	Age (years)	Functional class	BNP (pg/mL)	sPAP (mmHg)	dPAP (mmHg)	mPAP (mmHg)	CI (L/m/m^2^)	TPR (Dynes.s.cm^-5^)
1	Female	43	IV	305	91 (echo)	NA^a^	NA^a^	NA^a^	NA^a^
2	Male	70	III	243	62	21	40	2.5	615
3	Male	62	II	31	65	15	34	3.5	349
4	Male	72	III	21	66	32	43	2.87	447
5	Female	44	II	23	42	17	27	2.98	43

^a^No right heart catheterization due to right atrial mass

We explored cell-cell interactions based on ligand and receptor expression using Connectome^42^, which utilizes ligand-receptor data from FANTOM5^43^. First, we performed a centrality analysis to identify top ligand producers and correlating receptor receivers in signaling families from Connectome (Supplementary Fig. 3a). Myofibroblasts, endothelial cells, and foamy macrophages often displayed the most prominent outgoing and incoming centrality. To further investigate the endothelial cell niche, we explored communication pathways for each of the endothelial cell clusters (Supplementary Figs. 4, 5). The EC clusters displayed connectivity to a majority of cell types through both ligand and receptor interactions. The overall profile of communication pathways was unique to each EC cluster. There were both shared and unique ligands and receptors driving these communication pathways. These observations support the growing evidence of endothelial heterogeneity, even with organs^44^.

### Specific EC clusters in CTEPH may contribute to endothelial regeneration, inflammation and PH

To better characterize the EC populations in CTEPH, we subclustered the four EC clusters (clusters 8/9/11/12) that co-express CD34, CD31, and VWF and found seven endothelial clusters (Supplementary Fig. 6a). Genes expressed included markers related to endothelial repair and regeneration, inflammation, and TGF**β**-signaling (Supplementary Fig. 6b, Supplementary Data 1). EC.1 expresses marker genes linked to vascular repair and pulmonary hypertension. Among these, A2M, an inhibitor of fibrinolysis^45^, supports a role in CTEPH-related thrombosis. We also highlight SOX17 for its role in endothelial regeneration and known mutations in heritable PAH^46^. Inflammatory clusters EC.2, EC.3 and EC.5 express SELP (encoding P-selectin) as a marker gene, and EC.5 expresses VCAM1 and ICAM1 as marker genes, in keeping with a progression from the initial capture and rolling of leukocytes mediated by P-selectin to arrest and crawling mediated by VCAM1 and ICAM1^47^. Furthermore, EC.3 and EC.5 may contribute to PH through the expression of EDN1 as a marker gene in EC.3 and BMP6 as a marker gene in both EC.3 and EC.5^48^.

### CTEPH ECs display marked heterogeneity of phenotypes and express markers of both pulmonary artery and bronchial artery ECs

Intraoperative and pathologic examination of CTEPH lungs shows a connection between the bronchial circulation and neovessels within the fibrothrombotic CTEPH tissue^11^. Therefore, CTEPH ECs could originate from either the pulmonary or systemic circulation. Thus, the choice of pulmonary vs bronchial ECs as controls could confound the interpretation of differentially expressed genes in CTEPH specimens.

To address this possibility, we assessed the expression of marker genes for pulmonary artery ECs (PAECs) and bronchial artery ECs (BAECs) using classification from the Human Lung Cell Atlas^35,38^, which distinguishes at least six pulmonary EC subtypes. EC clusters from our PEA specimens display markers of both PAECs and BAECs (Supplementary Fig. 6c). Furthermore, there was a unique population of CTEPH ECs that co-express markers for both PAECs and BAECs within single cells. For example, E.6 displays mainly BAEC markers, whereas E.5 displays both PAEC and BAEC markers.

### CTEPH ECs have markedly altered phenotypes with both gain and loss of distinct subpopulations

As a result, we used both PAECs and BAECs from the Human Lung Cell Atlas^35^ as controls (Supplementary Table 3) to characterize EC subpopulations and identify aberrant gene expression patterns in CTEPH. To account for batch effects, variable genes were identified separately for control and CTEPH (Supplementary Fig. 7a) and then integrated for clustering analysis.

**Fig. 2:**
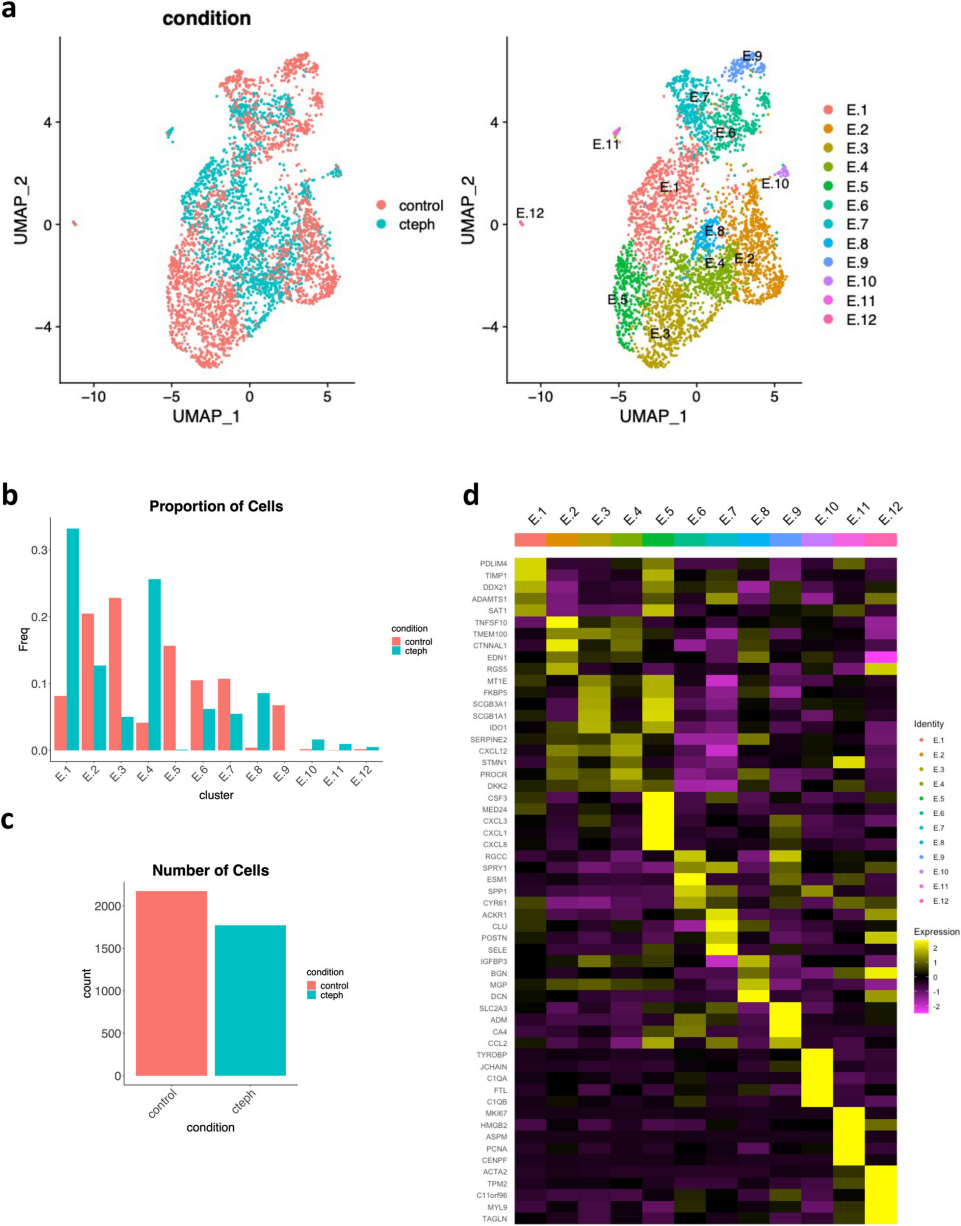
Differences in single-cell expression profiles between control and CTEPH endothelial cells. **a** UMAP visualization and clustering of endothelial cells from control and CTEPH. Left: UMAP visualization with cells parsed by control vs CTEPH. Right: UMAP visualization with 12 endothelial cell clusters shown. **b** Proportion of cells for each endothelial cell cluster in control and CTEPH. **c** Number of endothelial cells included in analysis for control and CTEPH. **d** Heatmap of top 5 markers for each endothelial cell cluster.

Twelve endothelial clusters were identified from this integrated dataset (Fig. 2a–c). The UMAP plot reveals distinct spatial distributions of cells from control and CTEPH, indicating unique transcriptional programs with gain and loss of EC subpopulations in CTEPH. E.1, E.4, and E.8 are mostly populated by CTEPH ECs, while E.2, E.3, E.5, E.6, and E.9 are mostly populated by control. Some clusters are almost exclusively represented by either control or CTEPH. For example, E.5 expresses PAEC markers and is almost exclusively populated by control ECs whereas E.8 co-expresses both PAEC and BAEC markers and is almost exclusively populated by CTEPH ECs (Fig. 2b,). Clusters with a higher proportion of CTEPH ECs often express mixed PAEC/BAEC markers. Using *propeller*^49^, we did not find statistical differences between control and CTEPH in terms of cell proportions for each cluster (Supplementary Fig. 7b, c), likely due to intersample heterogeneity that is common for scRNAseq^49^, and low cell numbers for a given cluster and sample.

Overall, marker genes were relatively specific for each cluster (Fig. 2d, Supplementary Data 2). Some clusters, such as E.5, have marker genes (e.g. CXCL8) that are highly restricted to the cluster whereas E.4 has marker genes (e.g. CXCL12, PROCR) that are also expressed across other clusters. To better understand the functional distinctions between clusters, we performed Gene Ontology (GO) analysis (Supplementary Fig. 8), as many clusters were predominantly composed of ECs from either control or CTEPH. Clusters enriched with CTEPH ECs were associated with GO terms related to hemostasis (E.1), angiogenesis and vascular development (E.4), smooth muscle proliferation and response to transforming growth factor (E.8), immune response and leukocyte activation (E.10), and the cell cycle (E.11) (Supplementary Fig. 9). In contrast, clusters with a higher proportion of control ECs showed enrichment in GO terms linked to endothelial development and copper response (E.3), regulation of inflammatory response (E.5), and positive regulation of cytokine production and mononuclear cell differentiation (E.9). We inferred transcription factor activity using DoRothEA^50^ and VIPER^51^ and found unique activity profiles across clusters (Supplementary Fig. 9). E.5 showed a strong activity for RELA and NFKB1, in keeping with GO terms for regulation of inflammatory response. Some clusters showed strong activity of HIF-1**α** (E.9, E.12). In some cases, shared TF activity between two clusters appears to coincide with proximity as seen on dimensionality reduction. For example, E.6 and E.7 both show strong activity of MEF2C and GATA6.

To ascertain whether current treatments for Group 1 PH that target EC pathways might apply to EC pathology in CTEPH PEA specimens, we assessed the expression of selected genes within the pathways for endothelin receptor antagonists, nitric oxide production, prostacyclin synthesis and Bone Morphogenic Protein Receptor 2 (BMPR2) signaling^52^. In CTEPH ECs, there was no obvious increase in endothelin-1 (EDN1) or the endothelin receptor type B (EDNRB), nor was there deficiency in expression of endothelial nitric oxide synthase (NOS3) or prostacyclin synthase (PTGIS) (Supplementary Fig. 10). Similarly, there did not appear to be deficiency in BMPR2 signaling, with increased expression of BMPR2 across clusters, and no obvious deficit in partnering receptors activin receptor-like kinase 1 (ACVRL1, also known as ALK1) or endoglin. While the RNA expression here does not clearly support the therapeutic activity of Group 1 PH therapies, there could still be therapeutic potential due to translational or post-translational changes in these targets that affect protein levels or activation (e.g. phosphorylation).

### Dysfunction of endothelial thrombotic regulation in CTEPH

The most populated cluster (E.1) included a greater number of CTEPH ECs compared to control and invoked multiple GO terms for hemostasis and coagulation (Supplementary Fig. 9). The natural history of resolving thrombosis progresses from thrombogenesis and fibrinolysis, to immune cell infiltration, and neovascularization of the thrombus through angiogenesis^5,20^. In contrast, evidence suggests that increased TGF-**β**-signaling with endothelial-mesenchymal transition is a feature of delayed thrombus resolution and pathologic thrombus organization in CTEPH^25,40^. Smooth muscle proliferation and response to TGF-**β** were also GO terms invoked by E.8, which had higher proportion in CTEPH ECs. Therefore, we evaluated these pathways to build a global picture of endothelial dysregulation through the stages of thrombus resolution in CTEPH.

**Fig. 3:**
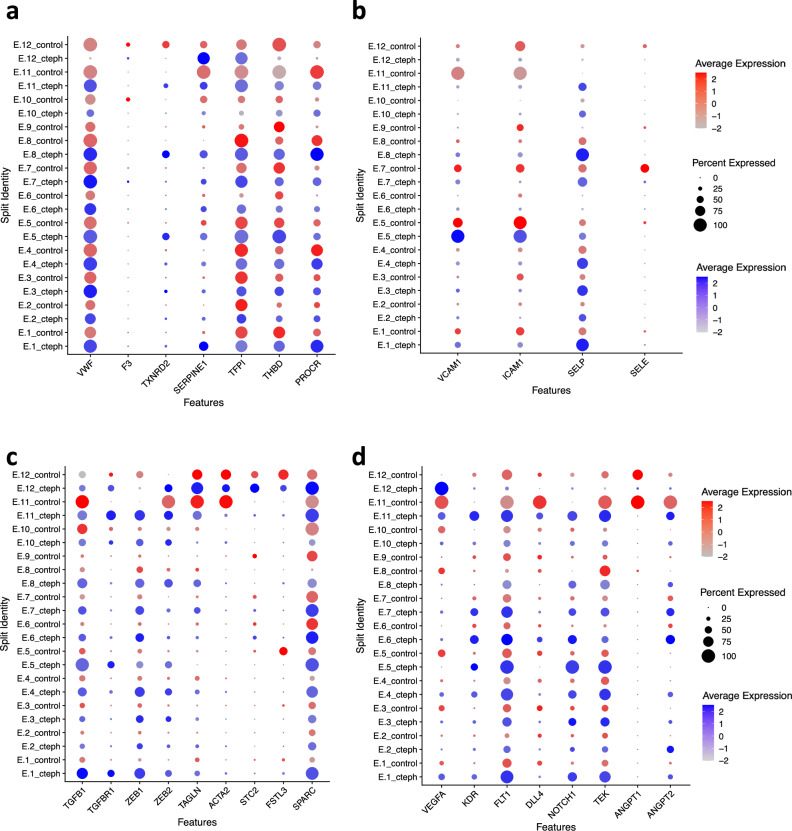
Endothelial dysregulation in thrombogenesis and thrombus resolution in CTEPH ECs. Dot Plots split by condition (control vs CTEPH) showing expression of markers of (**a**) endothelial thrombotic regulation, (**b**) endothelial activation, (**c**) TFG-**β** signaling, and (**d**) angiogenesis. Control ECs are represented in red and CTEPH ECs are represented in blue.

First, we evaluated markers of early endothelial thrombotic regulation (Fig. 3a)^20^. Von Willebrand factor (vWF) participates in both primary hemostasis and secondary hemostasis by activating platelets, initiating platelet aggregation, and by carrying coagulation factor VIII^20^. In keeping with evidence for epigenetic upregulation of vWF in CTEPH^39^, we found increased vWF RNA expression across the majority of endothelial clusters in CTEPH, including clusters from both PAECs (E.1, E.2, E.3, E.4) and BAECs (E.7) as well as clusters with control predominance (E.2, E.3, E.6) or CTEPH predominance (E.1, E.4, E.8). Two clusters, E.9 and E.12 showed the opposite trend, with greater vWF expression in control samples. These results demonstrate both quantitative and qualitative alterations in vWF regulation with a generalized trend of increased vWF expression yet selectively decreased expression in two clusters. Furthermore, there is a decrease in tissue factor pathway inhibitor (TFPI) (E.1-E.5, E.8), the major endogenous anticoagulant protein^53^. On the other hand, there is evidence for decreased fibrinolysis due to an increase in SERPINE1 (E.1, E.6, E.7, E.8, E.11, E.12), encoding the plasminogen activator inhibitor-1 (PAI-1), which is the principal inhibitor of tissue- or urokinase-type plasminogen activator^20^. Together, these observations reveal endothelial phenotypes that enhance coagulation and decrease fibrinolysis and are consistent with the presence of multiple stages of thrombosis seen in CTEPH^25^.

Because of the high immune cell content in CTEPH (Fig. 1), we asked whether CTEPH ECs contribute to persistent immune cell recruitment to the PEA tissue. SELP, which encodes P-selectin, is involved in the initial capture and rolling of leukocytes^47^ and platelets on activated endothelium^54^. SELP was a prominent EC receptor in our cell interaction analysis of CTEPH ECs (Supplementary Fig. 5). SELP expression was more frequent and higher in CTEPH than control ECs across multiple clusters (Fig. 3b). Interestingly, SELP expression was higher in CTEPH ECs in clusters with a higher proportion of CTEPH ECs (E.1, E.4, E.8, E.10, E.11) as well as in clusters with a higher proportion of control ECs (E.3, E.4)(Fig. 2b, Fig. 3b), suggesting a more generalized driver of SELP expression in CTEPH that can facilitate indiscriminate inflammatory cell recruitment. There is also a decrease in expression of VCAM1, ICAM1 and SELE in E.7 and E.11, in keeping with a dyscoordination of the specific subpopulations of ECs that can recruit leukocytes.

Recently, TGF**β**-signaling has been implicated in thrombus non-resolution and CTEPH^25,40^. Our results support increased TGF**β**-signaling with increased expression of TGFB1, TGFBR1, ZEB1, ZEB2 and SPARC across multiple clusters (Fig. 3c). We also found increased expression of STC2 in E.12^25^.

**Fig. 4:**
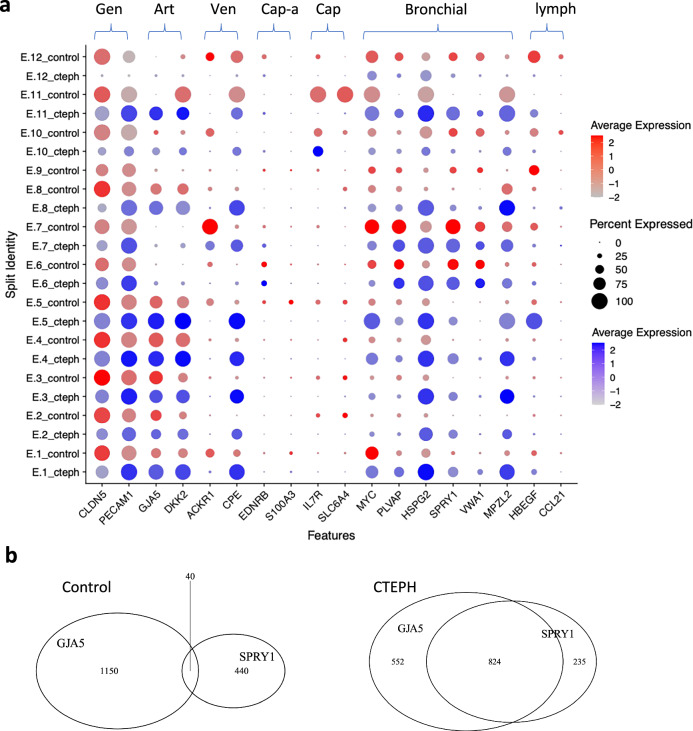
Individual ECs in CTEPH PEA specimens express both pulmonary artery (PA) and bronchial artery EC markers. **a** Dot plot showing expression of lung endothelial marker genes in control and CTEPH endothelial clusters. Gen, general endothelial cell marker; Art, PA; Vein, pulmonary vein; Cap-a, capillary aerocyte; Cap, general capillary cell; Bronchial, bronchial vessel; lymph, pulmonary lymphatic endothelial cell. **b** Venn diagrams showing expression of pulmonary arterial endothelial cell marker, GJA5, and pulmonary bronchial endothelial marker, SPRY1, in individual endothelial cells.

Angiogenesis is a critical process in thrombus resolution^22,24^, and defects in angiogenesis are implicated in CTEPH^23,41^. Sprouting angiogenesis relies on endothelial phenotypic diversity, with some ECs adopting either a “tip cell” morphology or a “stalk cell” morphology to generate a hierarchically branched and perfused vascular bed^37,55,56^. We note a decrease in the “tip cell” marker DLL4 in CTEPH ECs across a majority of endothelial clusters, except E.6, suggesting a decreased capacity to form “tip cells” in CTEPH (Fig. 4d)^55,57^. Along with an increase in NOTCH1 expression, which can be found in “stalk cells”, these observations suggest an imbalance of “tip” and “stalk” cell phenotypes in CTEPH ECs. Increased ANGPT2 can delay venous thrombus resolution and has previously been observed in CTEPH^41^. Our results confirm these findings and show widespread increase in ANGPT2 RNA expression in CTEPH, except E.11, where that trend is reversed.

Together, these observations reveal that CTEPH ECs display a transcriptional profile of endothelial dysfunction across the life cycle of thrombus generation and thrombus resolution.

### Cell populations that contribute to endothelial repair and regeneration differ between control and CTEPH ECs

The overall rate of endothelial cell replication is minimal in adult healthy blood vessels, and endothelial regeneration in large vessels is driven by a rare but “high proliferative potential” (HPP) subpopulation^58^. These ECs are characterized by activation of stress response genes, including ATF3 and are thought to be differentiated ECs adjacent to injury sites. There is a higher proportion of HPP ECs in control clusters, including PAEC (E.5) and BAEC (E.7, E.12) clusters (Supplementary Fig. 11a–c).

In addition to these differentiated ECs, vascular repair by ECs is also proposed to occur in large vessels through tissue-resident vascular endothelial stem cells (VESCs) that express CD200 and BST1^21^. We found a greater proportion of VESCs in CTEPH, concentrated in cluster E.11 (Supplementary Fig. 10d–f), in keeping with previous descriptions of putative endothelial progenitor cells in CTEPH^59^. These results suggest relative differences in endothelial regeneration pathways between control and CTEPH ECs with a shift in balance from HPP cells to VESCs.

### Individual ECs in CTEPH co-express both PAEC and BAEC markers

Clusters with a higher proportion of control ECs display single markers of anatomic origin, either PAEC (E.5) or BAEC markers (E.6)(Supplementary Table 4). In contrast, clusters with a higher proportion of CTEPH ECs tend to display mixed markers of both PAECs and BAECs. Within these clusters, it is the CTEPH ECs that show expression of both PAEC and BAEC markers while the control ECs almost exclusively express either PA or BA markers (Fig. 4a).

Clusters that show expression of both PAEC and BAEC markers could represent the presence of two converging populations of ECs, where expression of PAEC or BAEC markers occurs within different cells, or co-expression of both marker sets within individual cells. Therefore, we assessed the expression of PAEC and BAEC markers within individual cells (Fig. 4b). Control ECs almost exclusively expressed either the PAEC marker, GJA5, or the BAEC marker, SPRY1, in keeping with their selection from those populations^35^. In contrast, approximately half of the CTEPH ECs (824/1611) expressed both markers, suggesting a convergence between PAEC and BAEC phenotype in CTEPH ECs. Of note, approximately 15% (235/1611) of CTEPH ECs were BAECs (GJA5-, SPRY1 + ), which could be enough to confound gene expression analyses between CTEPH ECs if PAECs are the only control.

### Differences exist between control and CTEPH ECs, regardless of anatomic origin

**Fig. 5:**
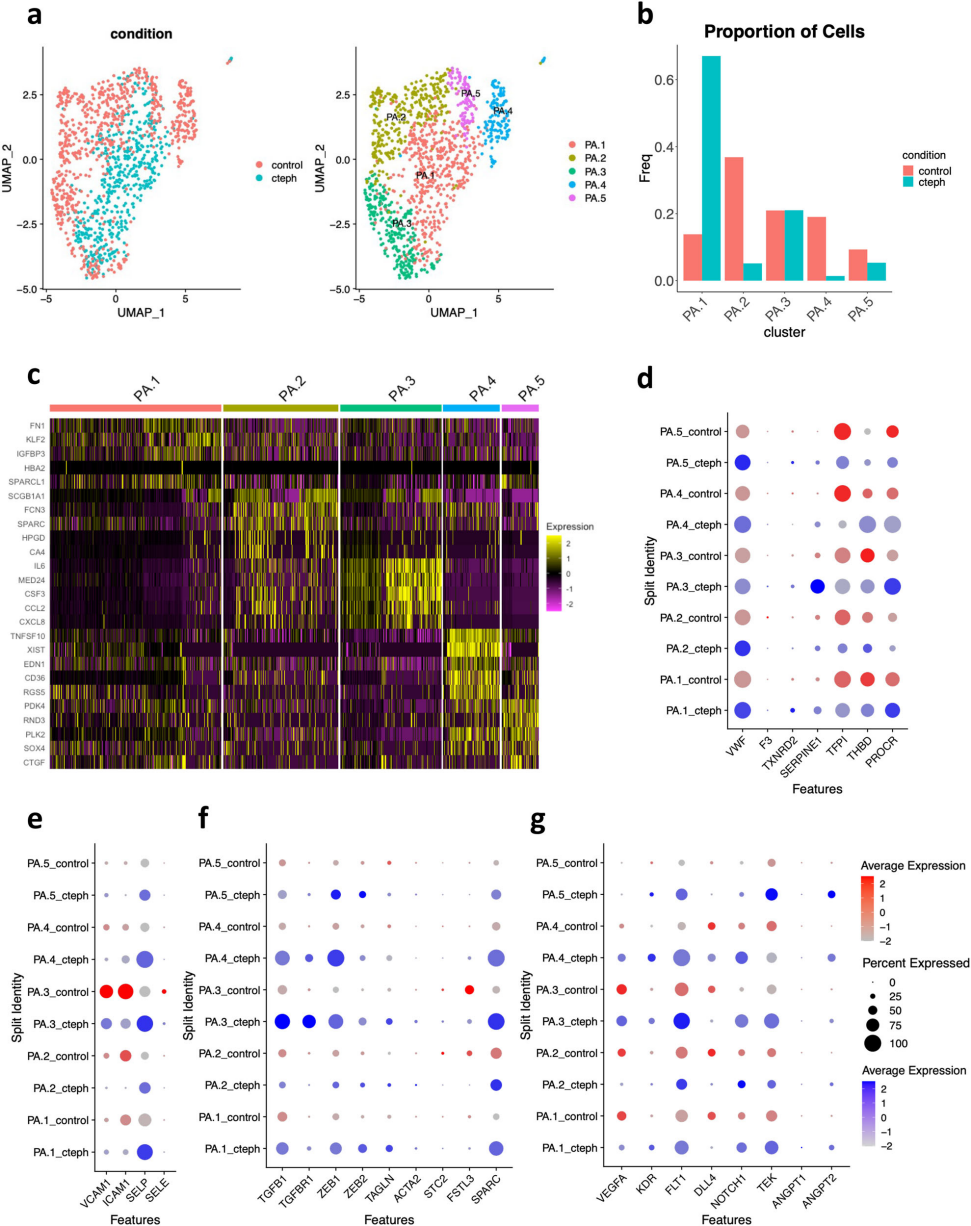
Analysis of gene expression in endothelial cells expressing PA endothelial markers exclusively. **a** UMAP visualization and clustering of endothelial cells expressing PA endothelial markers from control and CTEPH endothelial cells. Left: UMAP visualization with cells parsed by control vs CTEPH. Right: UMAP visualization with 5 PA endothelial cell clusters shown. **b** Proportion of PA endothelial cells for each cluster in control and CTEPH. **c** Heatmap of top 5 markers for each PA endothelial cell cluster. **d–g** Expression of markers of (**d**) endothelial thrombotic regulation, (**e**) endothelial activation, (**f**) TFG-**β** signaling, and (**g**) angiogenesis in PAECs. Control ECs are represented in red and CTEPH ECs are represented in blue.

“Pure” PAECs are rare in CTEPH PEA specimens. Subclustering with PAEC markers controls for alterations in EC phenotype and gene expression that may be confounded by the presence of BAEC within the PEA tissue. Subclustering of PAEC populations (GJA5/DKK2+, SPRY1-) revealed five clusters (Fig. 5a, b). A majority of CTEPH ECs clustered into PA.1, with few CTEPH ECs in PA.2 and PA.4. We found some expression of BAEC markers other than SPRY1 in both control and CTEPH (Supplementary Fig. 12a–c). However, when we attempted to negatively select for multiple BAEC markers, “pure” PAECs remained in the control group, but were almost absent in CTEPH. These findings further highlight the marked shift in the PAEC population in CTEPH.

We originally chose SPRY1 for this analysis because it was the most highly, frequently and specifically expressed of the BAEC markers in the Human Lung Cell Atlas^35^. Therefore, we used SPRY1 alone as a negative selection marker for the purposes of further analysis of PAECs.

Marker genes revealed clusters of ECs that show markers of laminar shear stress (KLF2, PA.1), inflammation (IL6, CCL2, CXCL8, PA.3), and female cells (XIST, PA.4)(Fig. 5c)^33,60,61^. Increased KLF2 in PA.1 is consistent with the possibility that CTEPH ECs with PAEC origin are exposed to higher laminar shear stress through bronchial artery collateralization. Within the PAEC subpopulation in CTEPH, we found similar perturbations in pathways affecting thrombogenesis, fibrinolysis, endothelial activation, TGF-β signaling and angiogenesis (Fig. 5d–g). We note changes to genes that are typically broadly expressed across all subclusters, such as VWF (Fig. 5d), as well as those that are expressed more selectively in control ECs, such as SELP (Fig. 5e). Similarly, there are shifts in PAEC populations that contribute to vascular repair, with a greater proportion of HPP ECs in the control group and a higher proportion of tissue-resident VESCs in the CTEPH group (Supplementary Fig. 13). Overall, these observations support altered PAEC phenotypes along the axis of thrombosis and thrombus resolution.

ECs from CTEPH often co-express markers of both PAECs and BAECs. Approximately half of CTEPH ECs co-expressed markers of both PAECs and BAECs in individual cells. In contrast, few control ECs displayed co-expression of PAEC and BAEC markers (Fig. 4). To compare control and CTEPH ECs which co-express both PAEC and BAEC markers in a “like-for-like” fashion, we sub-clustered this group of “double-positive” cells (GJA5+ & SPRY1+). Subclustering of this “double-positive” group of ECs revealed 6 clusters. While there were few control ECs in this analysis, they appeared across multiple clusters (Supplementary Fig. 14a–d). Differences between control and CTEPH still exist within this unique group of cells, even within clusters (Supplementary Fig. 15a–e). These subclustered ECs demonstrated patterns of dysregulated endothelial thrombotic regulation, with increased vWF, SERPINE1, SELP, TGFB1 expression and decreased DLL4 expression in CTEPH. Thus, changes in gene expression in CTEPH can be related both to the expansion of this “double-positive” subpopulation of ECs and to differences in gene expression within this group of ECs. However, given the low numbers of controls in this group, comparison of gene expression in this group should be interpreted with caution.

**Fig. 6:**
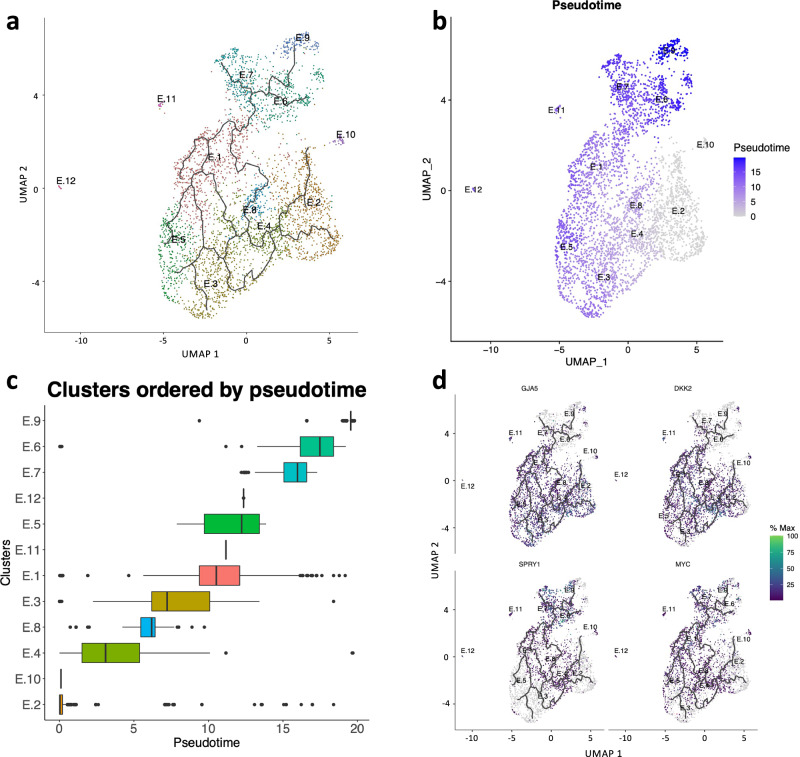
Trajectory analysis of endothelial cells. **a** Trajectory analysis overlaid on UMAP visualization of endothelial cell clusters. Multiple trajectories are indicated. **b** Pseudotime overlaid on UMAP visualization of clusters with E.2 (predominantly PA endothelial cells) used as a root for pseudotime analysis. **c** Clusters ordered by pseudo time with E.2 as a root. Note that E.2, E.3 and E.5 are “PA” endothelial cell clusters, E.6 and E.12 are “bronchial artery” endothelial cell clusters. **d** Examples of genes that are differentially expressed in pseudotime.

**Fig. 7:**
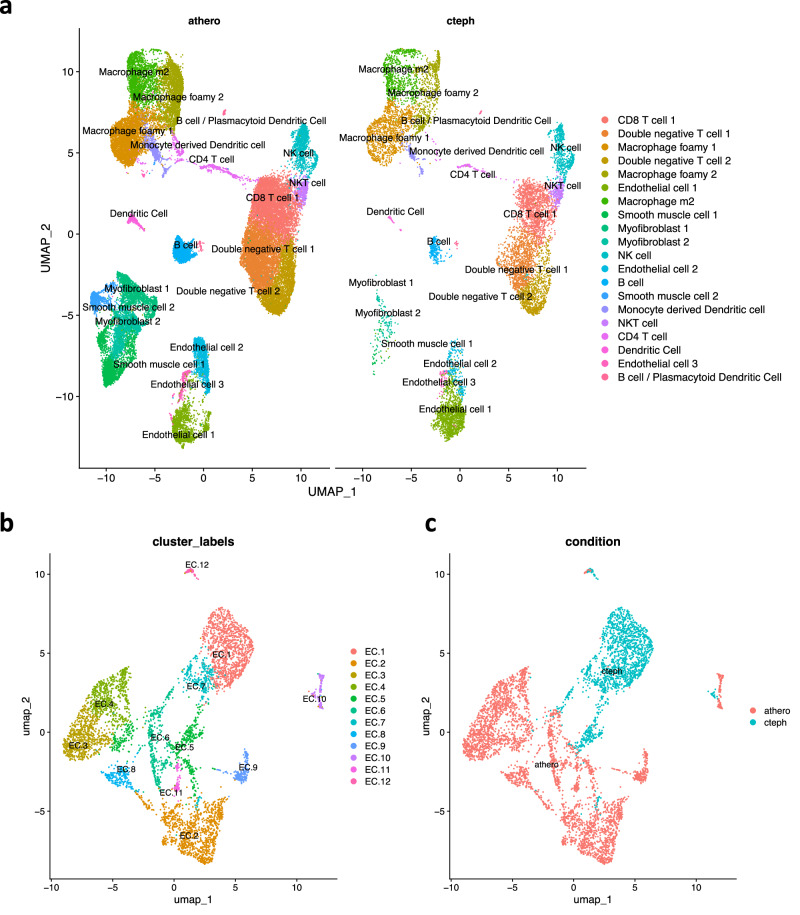
Comparison of CTEPH pulmonary endarterectomy specimens with atherosclerosis surgical specimens. **a** UMAP visualization and clustering of integrated single-cell RNA sequencing from atherosclerosis specimens (GSE159677) and pulmonary endarterectomy specimens. 20 cell populations are indicated. **b** UMAP visualization of subclustered endothelial cells. 12 endothelial subclusters are shown. **c** UMAP visualization of EC subclusters with cells parsed by atherosclerosis (athero) vs CTEPH.

Because this “double-positive” (mixed markers) population is almost exclusively present in CTEPH, we asked whether they originate from PAECs or BAECs. UMAP visualization of clustering suggests that some of these “double-positive” clusters (E.1, E.4, E.8) appear closer to PAEC clusters (E.2, E.3, E.5) than BAEC clusters (E.6)(Fig. 3a). To corroborate this observation, we performed trajectory analysis, revealing multiple trajectories within “double-positive” and PA clusters (Fig. 6a), whereas few trajectories were noted between “double-positive” clusters and the BAEC cluster E.6. Next, we ordered the clusters by pseudotime with E.2 (PAEC cluster) as a root and found that the “double-positive” clusters were ordered between the PAEC clusters E.2 and E.5, suggesting that these clusters may have origins from PAECs (Fig. 6b, c). Accordingly, the PAEC marker GJA5 and BAEC marker SPRY1 were differentially expressed in pseudotime (Fig. 7d).

Because bronchial collateralization in CTEPH^11^ would expose the nonresolved thrombus to arterial flows and oxygenated blood, we assessed if differences in hypoxia and shear stress from arterial vs. venous blood flow could contribute to these “double-positive” populations. We found that the hypoxia-responsive gene, VEGFA, was more highly expressed in PA and “double-positive” marker clusters (Supplementary Fig. 16) in keeping with lower oxygen content in the pulmonary circulation^37^. KLF2, a shear-stress responsive gene, appears to have higher expression in “double-positive” vs. PA clusters. Interestingly, NOS3/eNOS, which is responsive to both hypoxia and shear stress, appears to be most specifically expressed in “double-positive” clusters^33^. These data suggest that most “double-positive” clusters may originate from PAECs and that hypoxia and shear stress may contribute to these phenotypes.

Bronchial ECs harbor a population of activated ECs. We evaluated the group of ECs expressing pure BAEC markers (SPRY1 + , GJA5-). Subclustering revealed 5 clusters with UMAP visualization illustrating a shift in EC populations (Supplementary Fig. 17a–d, 18a). One cluster, BA.3, is characterized by inflammatory marker genes such as SELE, IL6, VCAM1, CSF3 and CXCL2 (Supplementary Fig. 17d), and is driven largely by control ECs (Supplementary Fig. 17c). Altered patterning of endothelial thrombosis regulation, thrombus resolution, and vascular repair also exist (Supplementary Fig. 18b–f) with some differences compared with PAEC subclustering (Fig. 5d–g). For example, there is proportionally more expression of TFPI in CTEPH subclustered BAECs whereas there is more TFPI in control subclustered PAEC. Similarly, there is not the same difference in DLL4 expression “tip” cells between control and CTEPH BAECs. Furthermore, control BAECs have a higher proportion of CD200 + BST1+ VESCs (Supplementary Fig. 18g), again highlighting the different phenotypes conferred by the infiltration of BAECs into PEA tissue.

### Compared to atherosclerosis, CTEPH PEA specimens display a similar immune profile and have distinct endothelial phenotypes

As suggested in the literature^12,13^, we compared single-cell RNA sequencing of CTEPH tissue with atherosclerosis tissue. Atherosclerosis is a vascular inflammatory disorder of the systemic circulation^62,63^. Overall, we found a similar complement of cell types between atherosclerosis (athero) from carotid endarterectomy (GSE159677) and CTEPH PEAs (cteph)(Fig. 7a, Supplementary Fig. 19). Some clusters were more highly populated by either CTEPH or atherosclerosis, with smooth muscle cell 2 and myofibroblast 1 showing statistically different proportions between the two conditions (Supplementary Fig. 20). Of note, the carotid endarterectomy specimens represent the full thickness of the vascular wall, whereas the CTEPH samples represent the endarterectomy specimen in isolation. This difference in sampling may account for differences in smooth muscle cell-derived cell types.

Next, we subclustered the ECs which revealed 12 EC clusters (Fig. 7b). The atherosclerosis and CTEPH ECs were generally spatially separated on UMAP visualization (Fig. 7c). As a result, we performed differential expression analysis after pseudobulking by disease state which revealed 1144 genes upregulated in atherosclerosis and 680 genes upregulated in CTEPH (Supplementary Fig. 21a). Some differences in EC phenotype are expected because of differences in vascular origin^30,31,33^. However, selected genes upregulated in CTEPH suggest differences between CTEPH and atherosclerosis pathophysiology. For example, increased expression of factor VIII (F8) is in keeping with increased thrombogenicity in CTEPH. We also found increased expression of the long noncoding RNA MEG8, which downregulates TFPI in ECs^64^. Interestingly, we found increased expression of PIEZO1 in CTEPH (Supplementary Fig. 21b). PIEZO1 is elevated in ECs from patients with pulmonary arterial hypertension (PAH) and inhibition of Piezo1 ameliorates experimental PAH in mice^65^. GO analysis revealed that biological processes increased in atherosclerosis often included aerobic respiration, in keeping with exposure to the systemic circulation. GO biological processes increased in CTEPH often included cell migration, in keeping with vascularization of the PEA specimen (Supplementary Fig. 21c). These data suggest differences in endothelial pathology between atherosclerosis and CTEPH that may impact the development of new therapies.

## Discussion

This study provides new insights into the pathophysiology of delayed thrombus resolution in CTEPH. Using scRNAseq, we uncovered remarkable heterogeneity of CTEPH ECs and reveal profound endothelial dysregulation (Supplementary Fig. 22). Assessment of gene expression directly from tissue without cell culture in this context facilitates these discoveries because lung ECs can lose their native phenotype in culture^38^.

The complexity of the cellular landscape and endothelial compartment within the CTEPH PEA specimen highlights the challenges in clarifying the pathophysiology and developing new therapies for patients ineligible for surgery. Coordinated action of intermingled EC subpopulations maintains physiologic homeostasis in alveolar capillaries, where ECs specialize into aerocytes for gas exchange and leukocyte trafficking, and general capillary ECs (gCaps) for stem/progenitor capacity in homeostasis and repair^34^. In this work, scRNAseq was critical to identify the appropriate EC subtypes for controls. We find that both pulmonary and bronchial artery ECs populate the CTEPH PEA specimen and discover a unique population of ECs co-expressing markers of both. These ECs appear more closely related PAECs upon dimensionality reduction and pseudotime analysis. The factors that contribute to these altered phenotypes remains a question.

To address the question of why there may be thrombus nonresolution in select cases of PE despite a paucity of blood-based coagulopathy^2,15,16^ in this population, we further assessed endothelial thrombotic regulation. We found disruption of multiple molecular pathways in CTEPH ECs, including clot initiation and fibrinolysis to subsequent dysregulated inflammatory responses, TGF-**β** signaling and angiogenesis. We confirm previously described alterations such as increased vWF expression, and SERPINE1 (encoding PAI-1 protein^17,18^), and also find decreased TFPI, a major endothelial anticoagulant^53,64^.

Furthermore, we identify SELP (encoding P-selectin) dysregulation in CTEPH. The endothelial inflammatory response is a tightly regulated system. For example, a subpopulation of ECs is epigenetically primed to produce VCAM1 in a graded response to Tissue Necrosis Factor-**α**^36^. In CTEPH ECs, we found both increased SELP expression levels and SELP expressed across a higher percentage of cells. Thrombus resolution includes a sequential recruitment of leukocytes^5^ which can be disrupted by indiscriminate expression of inflammatory adhesion molecules such as P-selectin.

Of therapeutic interest, P-selectin is also involved in other disorders of thrombosis. Upregulation of P-selectin contributes to the pathogenesis of vaso-occlusive crises in sickle cell disease^66^. Treatment with crizanlizumab reduces the frequency of these thrombotic events and is an approved therapy^66^. Blocking P-selectin also enhances thrombus resolution in a model of thrombosis using inferior vena cava ligation^67^. Blocking P-selectin reduces thrombus density and accelerates fibrin degradation, in keeping with an increase in urokinase-type plasminogen activator levels^67^. This balance of fibrinolysis is relevant in CTEPH ECs as we found increased SERPINE1, which encodes the plasminogen activator inhibitor-1 (PAI-1)^68^. Futhermore, blocking P-selectin reduced both platelet monocyte aggregates and and infiltration of thrombus by neutrophils and macrophages^67^. Thus, P-selectin presents an opportunity to address both local coagulopathy and immune-mediated antagonism of thrombus resolution. Beyond crizanlizumab, other inhibitors of P-selectin are under development^69,70^.

In addition to the endothelial dysfunction early in the evolution of thrombosis with altered coagulation, fibrinolysis and inflammation, we also find endothelial dysfunction later in the course of thrombus evolution with defective angiogenesis and endothelial-mesenchymal transition (TGF-**β** signaling). Recanalization of thrombus through angiogenesis may be inhibited in CTEPH by ECs with reduced “tip cell” potential^57,71^. How the “tip” to “stalk” ratio and coordination can be reset in CTEPH remains to be determined, and is a future direction to address in both CTEPH development and established CTEPH. Whether all of these altered domains of thrombus evolution need targeting will be an important question. Pathologic identification of multiple concurrent stages of thrombus organization from fresh thrombi to fibrosis in PEA specimens^25^, are in keeping with this possibility.

To date, the pathophysiology of CTEPH has remained elusive, especially without animal models that recapitulate human disease^40^. Histologic examination and scRNAseq have revealed the cellular complexity of the CTEPH PEA specimen^12,13^. We found similar cell types, though there were proportionally fewer fibroblasts and smooth muscle cells in our samples and a higher proportion in the lymphocyte and myeloid lineage. These differences could relate to differences in patient population and/or tissue dissociation protocols^72^. Heterogeneity and disease subphenotypes are an important consideration as the pathway to thrombus nonresolution may be variable between individuals, as with other lung disease^73^. Identifying the most therapeutic pathway for individual patients remains an important challenge.

Overall, these observations support previous lines of investigation in CTEPH that span multiple processes in thrombus generation and thrombus resolution and expand the picture of profound EC dysfunction in CTEPH. In isolation, evidence suggests that intervening on a single pathway (e.g. TGF-**β** signaling, P-selectin, ANGPT2) can modulate thrombus resolution. Whether this approach to treatment is sufficient for CTEPH and the best timing for each potential therapy remains to be determined. However, this data provides new insights into delayed thrombus resolution in CTEPH across multiple domains of thrombus evolution that may be assessed for therapeutic potential.

## Methods

### Tissue collection

PEA specimens were collected from CTEPH patients who underwent PEA in our institution from March–April 2019(Table 1)^4,10^. Samples were excluded if they did not meet quality control metrics for scRNAseq. The institutional ethics committee approved the protocol (University Health Network, REB# 18-5892). All patients signed informed consent prior to surgery.

### Single-cell isolation

Fresh PEA tissue was washed with HBSS to remove any non-resident cells. The tissue was then cut into 3 × 3 mm pieces and 4.5 ml HBSS buffer + 200 µL Collagenase A (10 mg/mL) + 80 µL DNAse (10 mg/mL) were added and digested in GentleMACS at 37 °C for 7 min after which the stopping buffer was added. Following collagenase treatment, the stop solution 10% FBS in HBSS was added into the tube and 40 mm filter was used to collect the dissociated single cells. The process was repeated by running GentleMACS with collagenase/DNAse buffer for another 7 min at 37 °C and filtered through 40 µm cell strainer. The mixed cells were then incubated with RBC lysis buffer for 5 min and washed in HBSS and purified by centrifugation. Protein alias is described in parentheses if different from gene name.

### Human Lung Cell Atlas

Data from non-disease (control) lung single-cell RNA sequencing were obtained from the Human Lung Cell Atlas (https://www.synapse.org/#!Synapse:syn21560554 downloaded June 21, 2021). These data included three samples (two male, one female)(Supplementary Table 3) of pathologically confirmed normal tissue as described in Travaglini et al.^35^.

### 10x sample processing and cDNA library preparation

Samples were prepared using the 10x Genomics Single Cell 3′ v2 Reagent Kit (please see 10x Genomics online, https://www.10xgenomics.com). The samples were washed twice with PBS plus 0.04% BSA (Life Technologies; Sigma). Sample viability was assessed via Trypan Blue (Thermo Fisher) and the samples with over 80–95% cell viability were processed. Each sample was processed to reach a target capture of 6000 cells and was pelleted, re-suspended and loaded onto the 10x Genomics single-cell-A chip. After droplet generation, samples were transferred onto a pre-chilled 96-well plate (Eppendorf), heat-sealed and reverse transcription was performed using a Veriti 96-well thermal cycler (Thermo Fisher). The Purified cDNA was amplified for 12 cycles before being cleaned up using SPRIselect beads (Beckman). Samples were diluted 4:1 (elution buffer (Qiagen):cDNA) and run on a Bioanalyzer (Agilent Technologies) to determine cDNA concentration. cDNA libraries were prepared by the Single Cell 3′ Reagent Kits v2 with appropriate modifications to the PCR cycles based on the calculated cDNA concentration (10X Genomics).

### Sequencing analysis

#### Processing Pipeline

The raw FASTQ files were aligned to the appropriate genome (hg19) using the STAR aligner (STAR v2.5.2b), which aligns reads simultaneously to the genome and the transcriptome. Both types of alignments were used in order to accurately determine whether a read could be confidently associated with a transcript and/or a gene. Accessory programs for the alignment stage include SAMTOOLS (v1.3.1) and BEDTOOLS (v2.26.0). The CELLRANGER (v3.0.2) pipeline was used to obtain two types of gene-barcode matrices. The first matrix is an unfiltered gene-barcode matrix. The filtered matrix contains every barcode from the fixed list of known barcode sequences. The matrix was loaded into R (v4.4.0) for the final graphical output of results and statistical analysis. The main tools used for the secondary analysis steps were: SCATER (v1.2.0), CELLRANGERRKIT (v1.1.0), SCRAN (v1.2.2), RTSNE (v0.11), SC3 (v1.3.14), EDGER (v3.16.5), SEURAT (v5.0.0), and PCAMETHODS (v1.50.0).

### Statistics

No statistical methods were used to predetermine sample size. Samples were integrated using shared highly variable genes to reduce batch effects and improve clustering. Briefly, variable genes for each condition were determined separately and shared variable genes were used for integration using canonical correlation analysis in SEURAT(v5.0.0). Principal component analysis, clustering, differential gene expression (using “FindAllMarkers” or “FindMarkers”), universal manifold approximation and projection visualizations, and trajectory analysis were performed using R (v4.4.0) and R packages: SEURAT (v5.0.0)^74^, Monocle3^75^. Cell interaction analysis was performed using Connectome(v1.0.0)^42^. Differential expression analysis of pseudobulked cells was performed with DESeq2(3.2)^76^ and GO analysis was performed using clusterProfiler(3.2)^77^ using the org.Hs.eg.db(3.2) database. Statistical testing of differential cell proportions was performed using Propeller^49^. Transcription factor activity was inferred using DoRothEA(1.18.0)^50^ and VIPER^51^.

We used two methods to determine the number of principal components to use for clustering analysis. First, we generated an elbow plot to visualize the standard deviation of each principal component to visualize the number of principal components where the standard deviations begin to plateau. Next, we calculated two values and chose the lower of the two to determine the number of principal components: (1) the range where principal components cumulatively contribute 90% of the standard deviation and further principal components contribute 5% of the standard deviation; (2) The point where percent change in variation between principal components is less than 0.1%.

## Supplementary information

Supplementary Figures v1.7b May 6 2025

Supplementary Data 1

Supplementary Data 2

## Data Availability

Full de-identified sequencing data for PEA specimens are available in the gene expression omnibus (GEO) under accession number GSE271435.
