# Supplementary material for: Distinct endothelial cells in chronic thromboembolic pulmonary hypertension
Source: NPJ Cardiovasc Health. 2025 Jul 2;2:33. doi: 10.1038/s44325-025-00072-8 (PMC12912402; doi:10.1038/s44325-025-00072-8)
Supplement: Supplementary file 1 — Supplementary Figures v1.7b May 6 2025 [file 44325_2025_72_MOESM1_ESM.pdf]

Sample	Estimated Number of Cells	Number of Sequencing Reads	Sequencing Saturation (%)	Median UMI Counts per Cell	Median Genes per Cell	Total Genes Detected
**288**	4268	115287679	31.8	4530	1624	21935
**289**	3556	116792088	49.8	3629	1394	21180
**292**	989	103999762	76.7	4714	1620	19792
**293**	2651	93025527	60.2	3658	1298	20449
**298**	1134	142180977	81.7	1502	590	17933

Supplementary Table 1. Cellranger output for CTEPH samples.

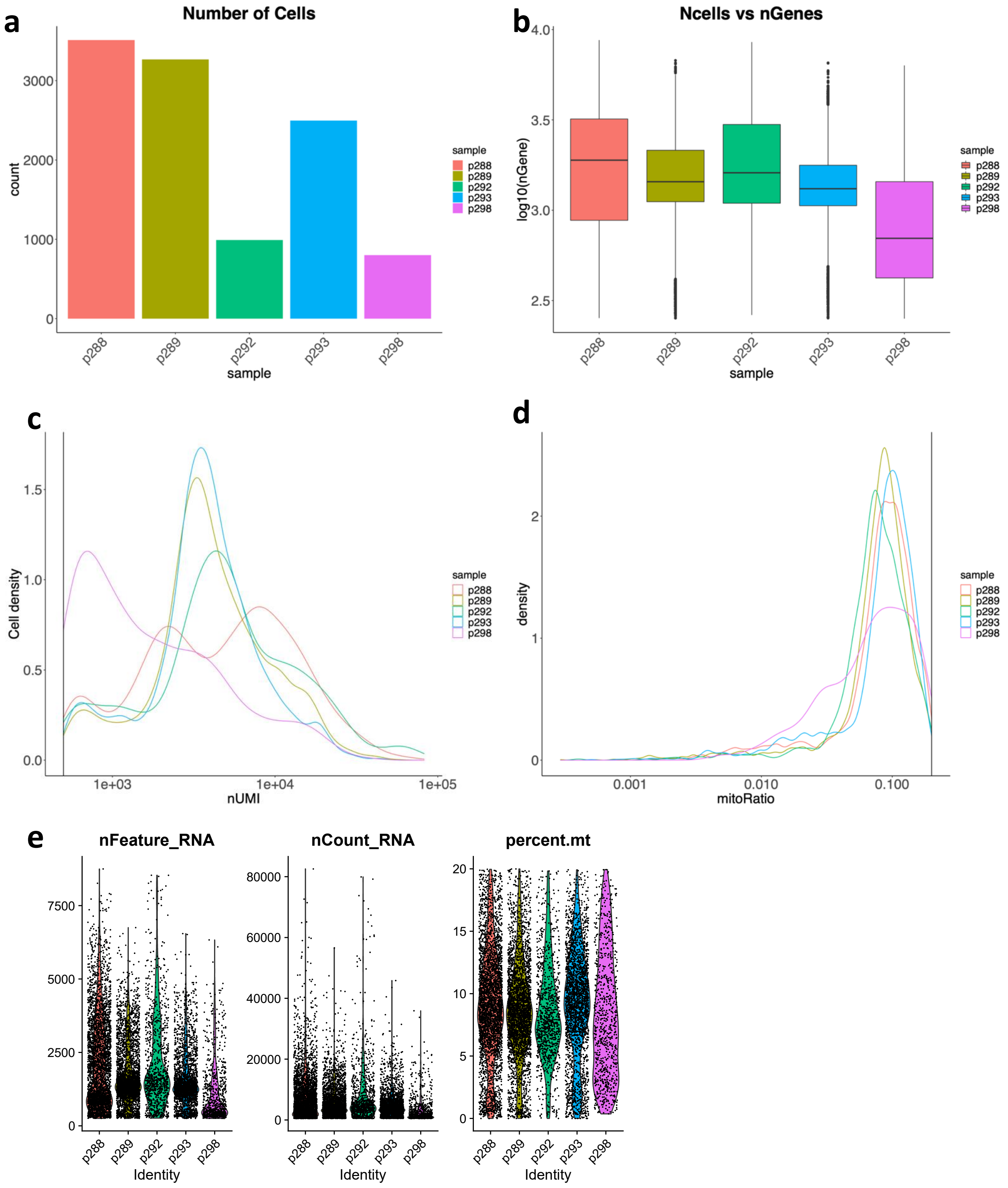

Supplementary Figure 1. Characteristics of CTEPH samples. **a)** Bar graph showing number of cells per sample. **b)** Boxplot showing number of genes per sample. **c)** Ridge plot showing number of unique molecular identifiers (nUMI) per sample. **d)** Ridge plot showing ratio of mitochondrial genes (mitoRatio) per sample. e) Violin plot representations of quality control metrics from Seurat.

Cell type	Positive marker	Negative marker
**Endothelial cell**	CD34, VWF, CD31, CDH5	
**Smooth muscle cell**	ACTA2, CNN1	
**B cell**	CD79A, MS4A1, CD19	
**Natural Killer**	CD94/KLRD1, CD122, NKG2D/KLRK1, CD161/KLRB1, CD56	CD3-
**Monocyte classic**	CD14++, CCR2	FCGR3A
**Monocyte intermediate**	CD14 ++, FCGR3A+, CX3CR1 high, CCR5+	
**Monocyte Non-classical**	CD14+, CD16 ++/FCGR3A, , CX3CR1 high	CCR2 low
**Macrophage**	CD14, CD68	
**Macrophage m1**	CD14, CD86, CD80, CD68, MHCII, IL-1R, TLR2, TLR4, iNOS,	
**Macrophage m2a**	CD14, CD163, SR, MMR/CD206/MRC1, CD200R	
**Macrophage m2b**	CD14, CD86, MHCII	
**Macrophage m2c**	CD163, TLR1, TLR8	
**Macrophage m2d**	VEGF	
**Platelet resting**	PPBP, CD42b/ - no GP1BA	
**Platelet activated**	SELP	
**Dendritic cell**	FCER1A, CST3	
**Conventional dendritic cell 1**	CD8A, CLEC9A, ITGAE	
**Conventional dendritic cell 2**	CD1C, CD207, ITGAM	
**Plasmacytoid dendritic cells**	CLEC4C/CD303, LILRB4, NRP1	
**Monocyte-derived dendritic cells (mo-DC)**	CD14, MRC1 (CD206), CD209	
**Natural Killer T-cell**	CD161/KLRB1 (typical of NK), [CD25/IL2RA, CD44, CD69	
**CD8 + T cell**	CD3D, CD8A	
**CD4 + T cell, naive**	CD3D, CD4, CD45RA/PTPRC, CCR7	
**CD4+ T cell, central memory**	CD3D, CD4, CCR7+, CD62L/SELL	CD45RA/PTPRC
**CD4+ T cell, effector memory**	CD3D, CD4	CCR7
**CD4+ T cell, tissue-resident memory**	CD103+/ITGAE, CD69+	
**CD4+ T cell, follicular helper**	CXCR5+, ICOS+, BCL6	
**CD4+ T cell, Th1**	CXCR3+, T-bet/TBX21	
**CD4+ T cell, Th2**	CRTH2+/PTGDR2, GATA3	
**CD4+ T cell, Th9**	PU.1/SPI1	
**CD4+ T cell, Th17**	CCR6+, CD161+, RORC2	
**CD4+ T cell, Treg**	FOXP3	CD25-/IL2RA, CD127-/IL7R,
**CD4+ T cell, type 1 regulatory**	CD49b+/ITGA2, LAG3+	CD25-, CD127-
**Mesodermal Killer**	CD16/FCGR3A, CD96, CD112/NECTIN2, CD137L/TNFSF9, FasL/FASLG, TRAIL/TNFSF10,	CD56-
**Mast cell**	TPSB2, FCER1A	
**Neutrophil**	CD15/FUT4, CD16/FCGR3A,	CD49d-/ITGA4
**Eosinophil**	CD11b/ITGAM, CD193/CCR3, EMR1/ADGRE1, Siglec-8/SIGLEC8	
**Basophil**	2D7 antigen, CD123/IL3RA, CD203c/ENPP3, FCER1A	CD117-/KIT
**Red blood cell**	CD235a	
**Fibroblast**	FSP1/S100A4, PDGFRA, COL1A1	
**Myofibroblast**	ACTA2, Vimentin, PALLD	

Supplementary Table 2. Gene markers used for cell type annotation.

a

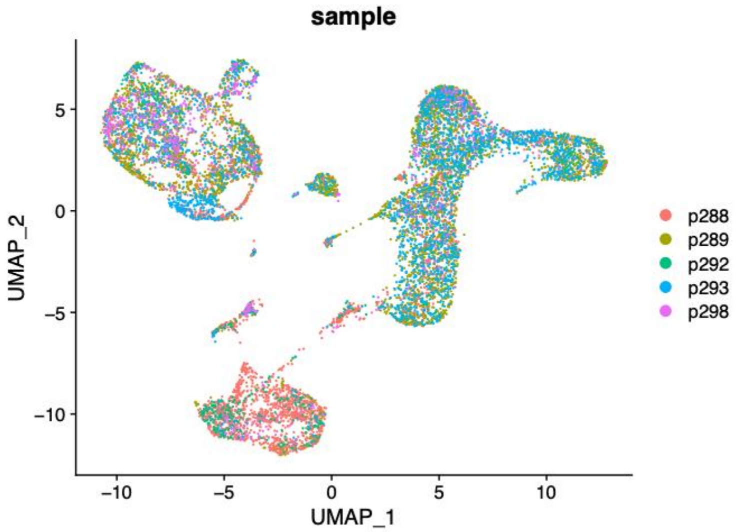

b

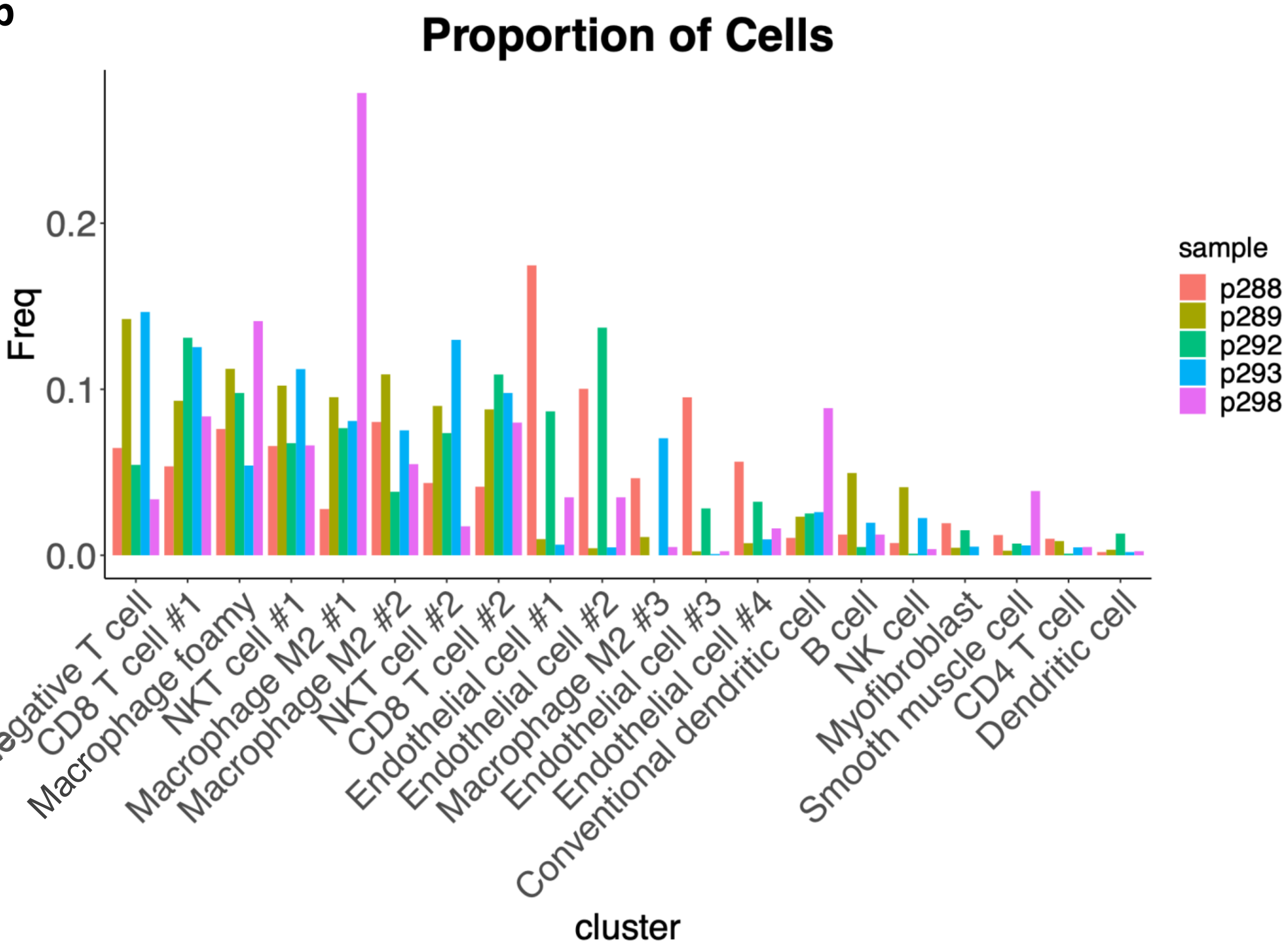

Supplementary Figure 2. Cell populations in CTEPH samples. **a)** UMAP visualization of cell clusters in CTEPH samples coloured per patient sample. **b)** Bar graph showing proportion of cells from each cluster per patient sample.

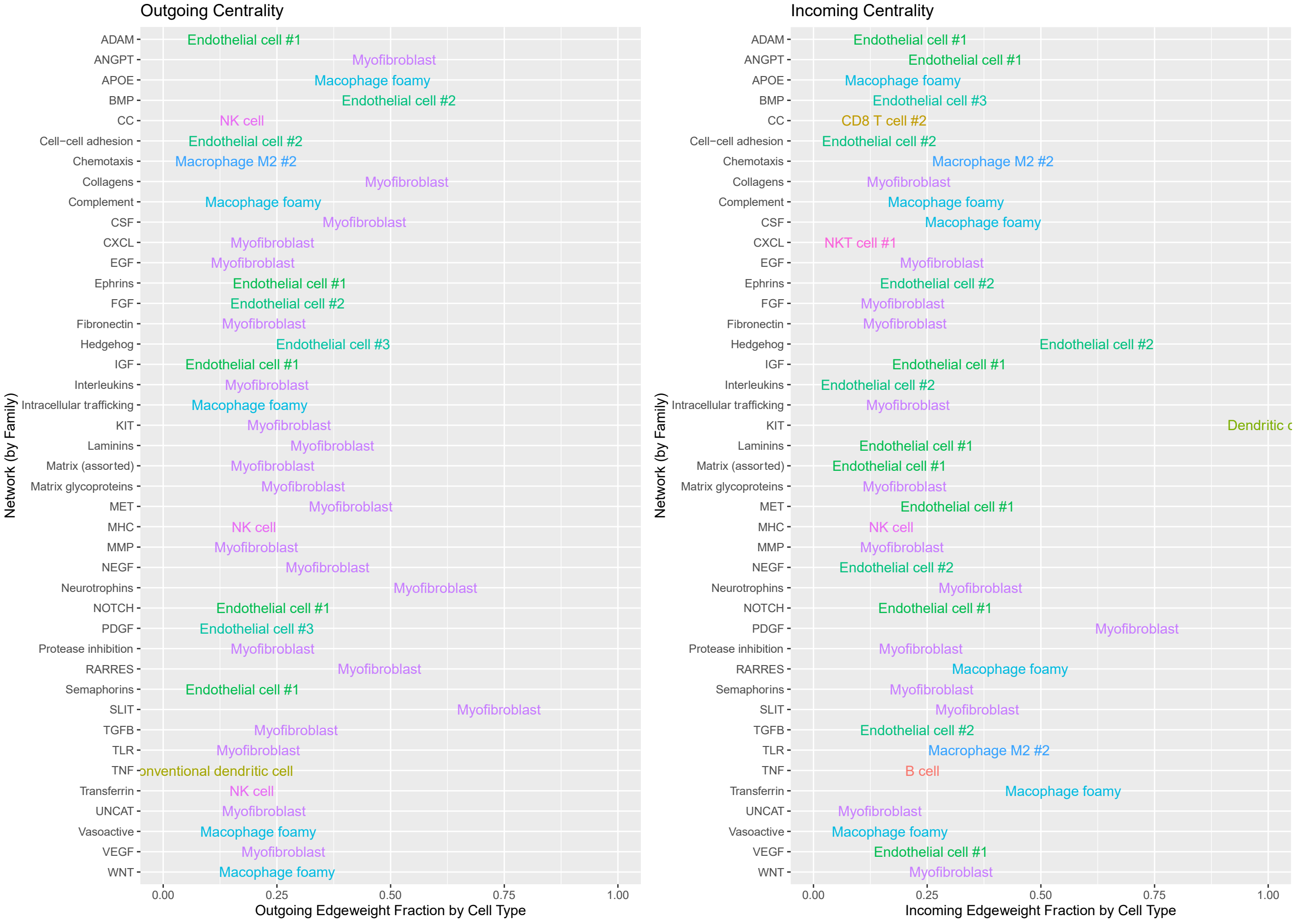

Supplementary Figure 3. Centrality analysis by signalling networks using Connectome. Outgoing centrality indicates clusters that are ligand producers for a given network, indicated on the y-axis. Incoming centrality indicates clusters that express correlating receptors for a given network.

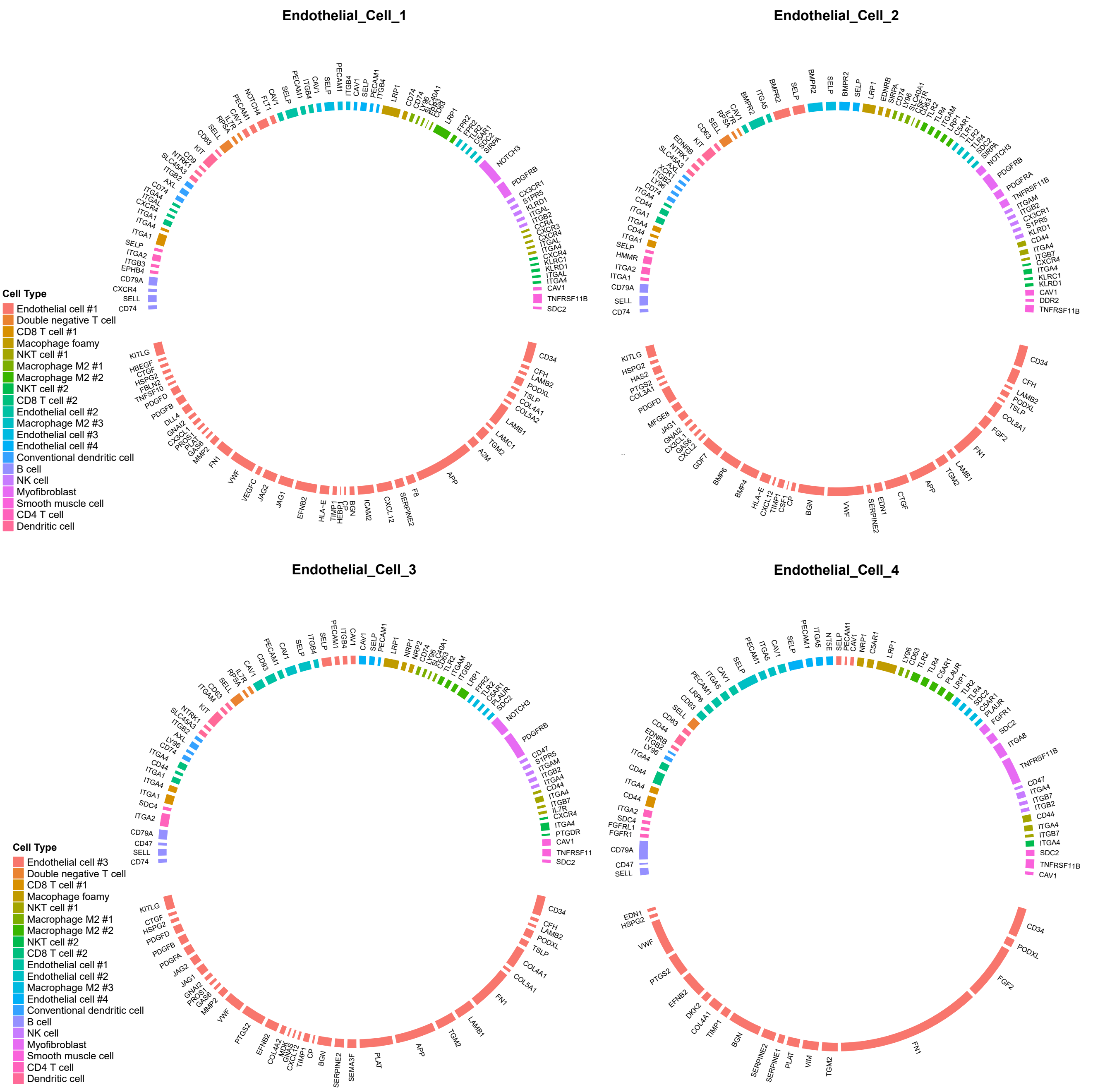

Supplementary Figure 4. Circos plots showing outgoing communication pathways (endothelial ligand expression) converging on endothelial cell clusters.

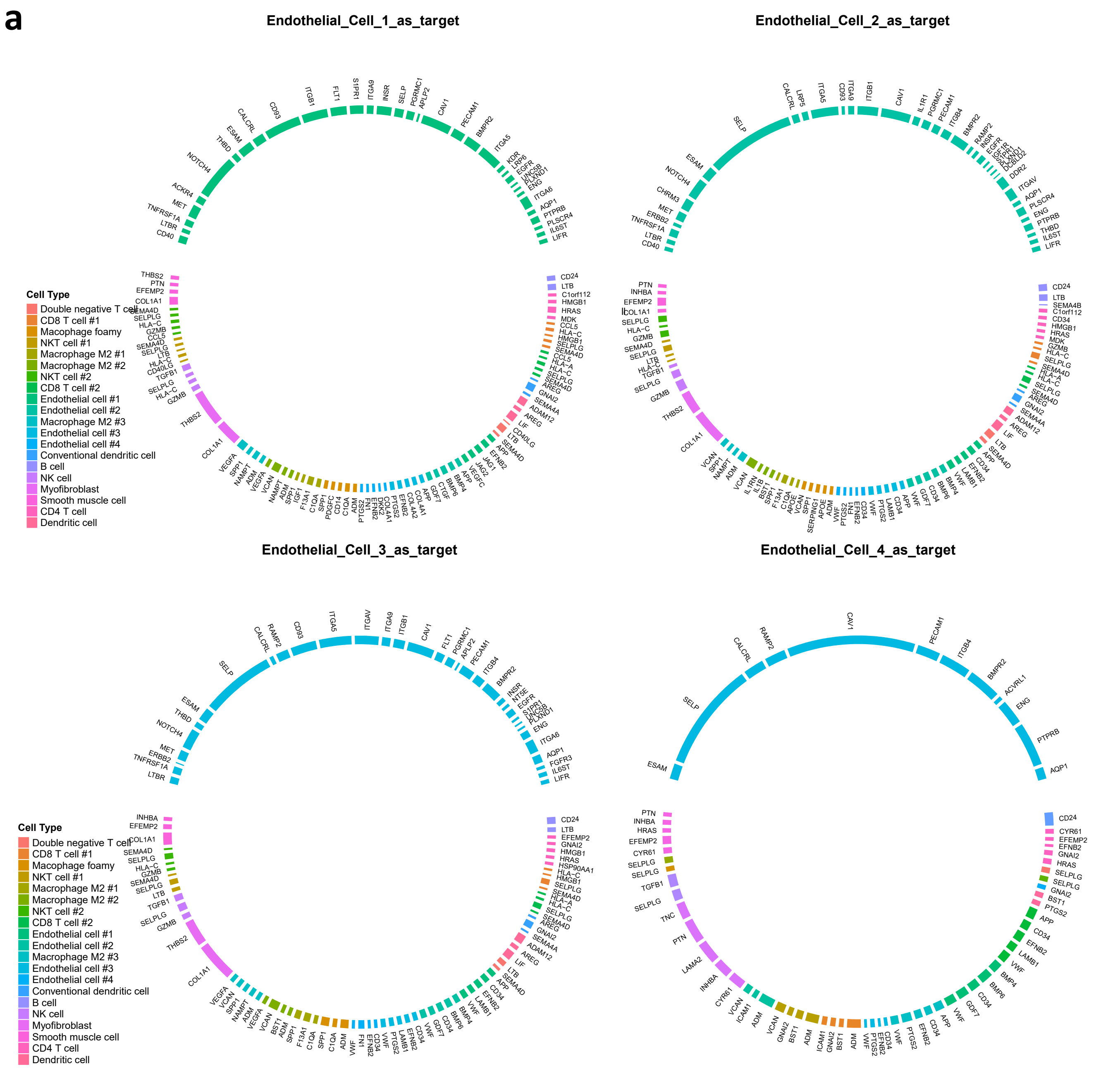

Supplementary Figure 5. Circos plots showing incoming communication pathways (endothelial receptor expression) converging on endothelial cell clusters.

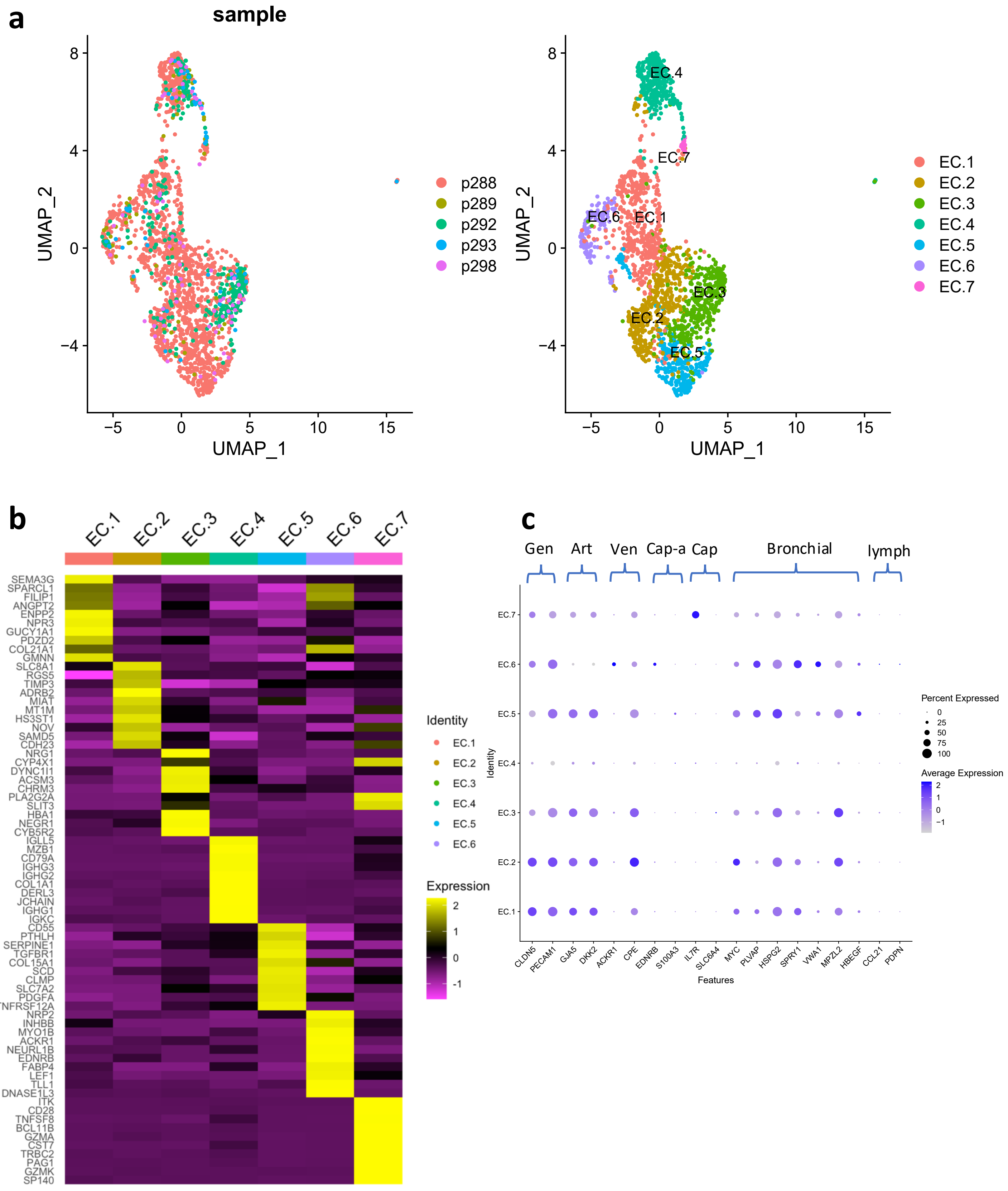

Supplementary Figure 6. Subclustering of endothelial cells from CTEPH specimens. **a)** UMAP visualization and clustering of endothelial cells from CTEPH by sample (left) and cluster (right). **b)** Heatmap of top 5 markers for each endothelial cell cluster from CTEPH. **c)** Dotplot of expression of pulmonary endothelial cell markers in CTEPH endothelial cell clusters.

Sample	Age	Sex	Diagnosis	Smoking status
**#1**	75	Male	Adenocarcinoma	Remote
**#2**	46	Male	Carcinoid	non-smoker
**#3**	51	Female	Carcinoid	non-smoker

Supplementary Table 3. Characteristics of control samples from human lung cell atlas.

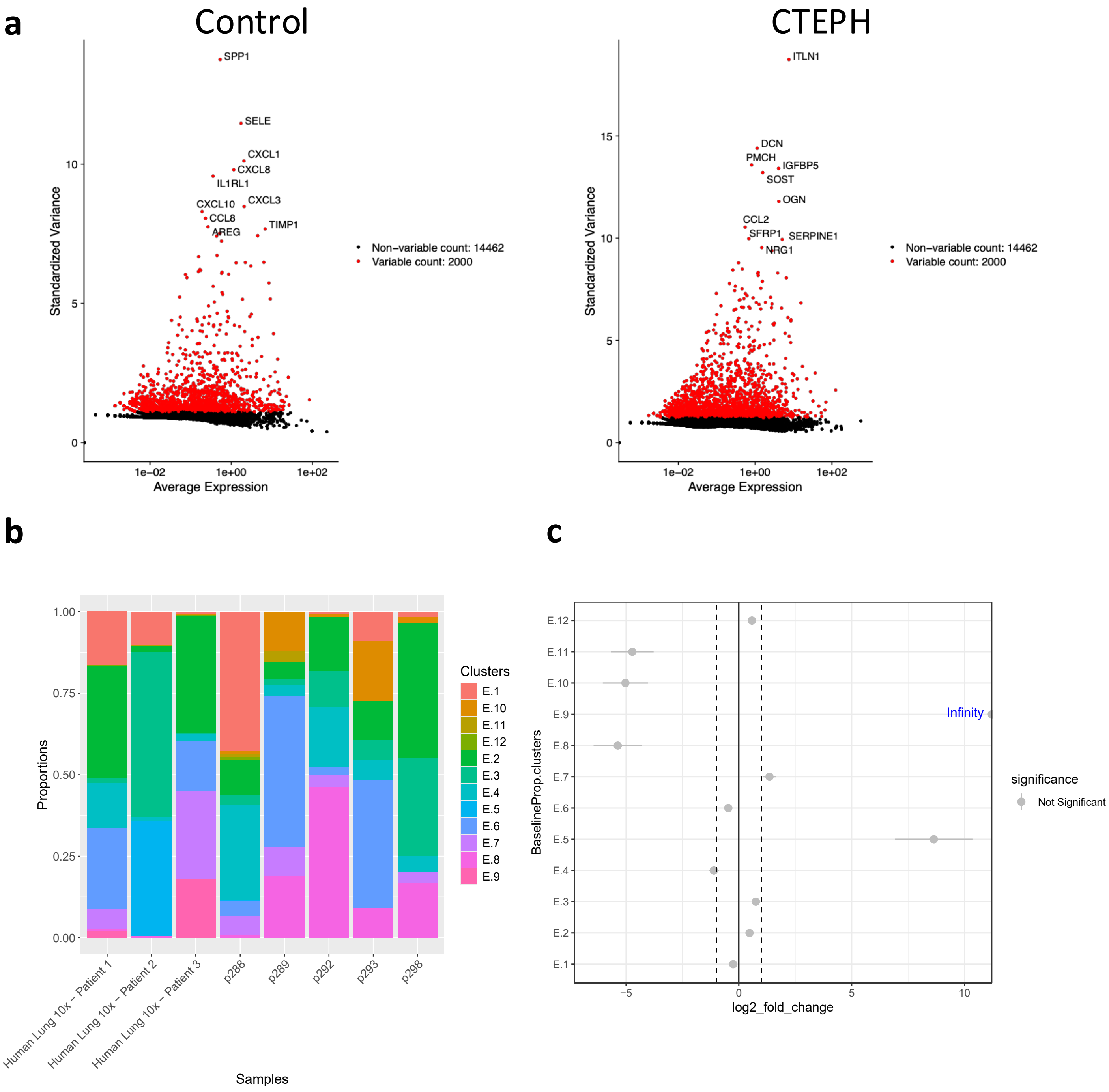

Supplementary Figure 7. Comparison of control and CTEPH ECs. **a)** Identification of variable features (genes) in control and CTEPH endothelial cells. **b)** Bar graph showing cluster proportions by sample. **c)** Gene Statistical analysis of cell proportion using *propeller*. Positive log2_fold_change indicates higher proportion of cells in control and negative log2_fold_change indicates higher proportion of cells in cteph. To account for heteroskedastic variance, propeller implements two transformations to stabilize variance prior to testing statistical differences using a linear modelling framework. Therefore, propeller does not report variance and the displayed error bars represent 20% of log2_fold_change. In this visualization, a red colour would indicate statistical significance, but no clusters showed statistically significant differences between control and cteph.

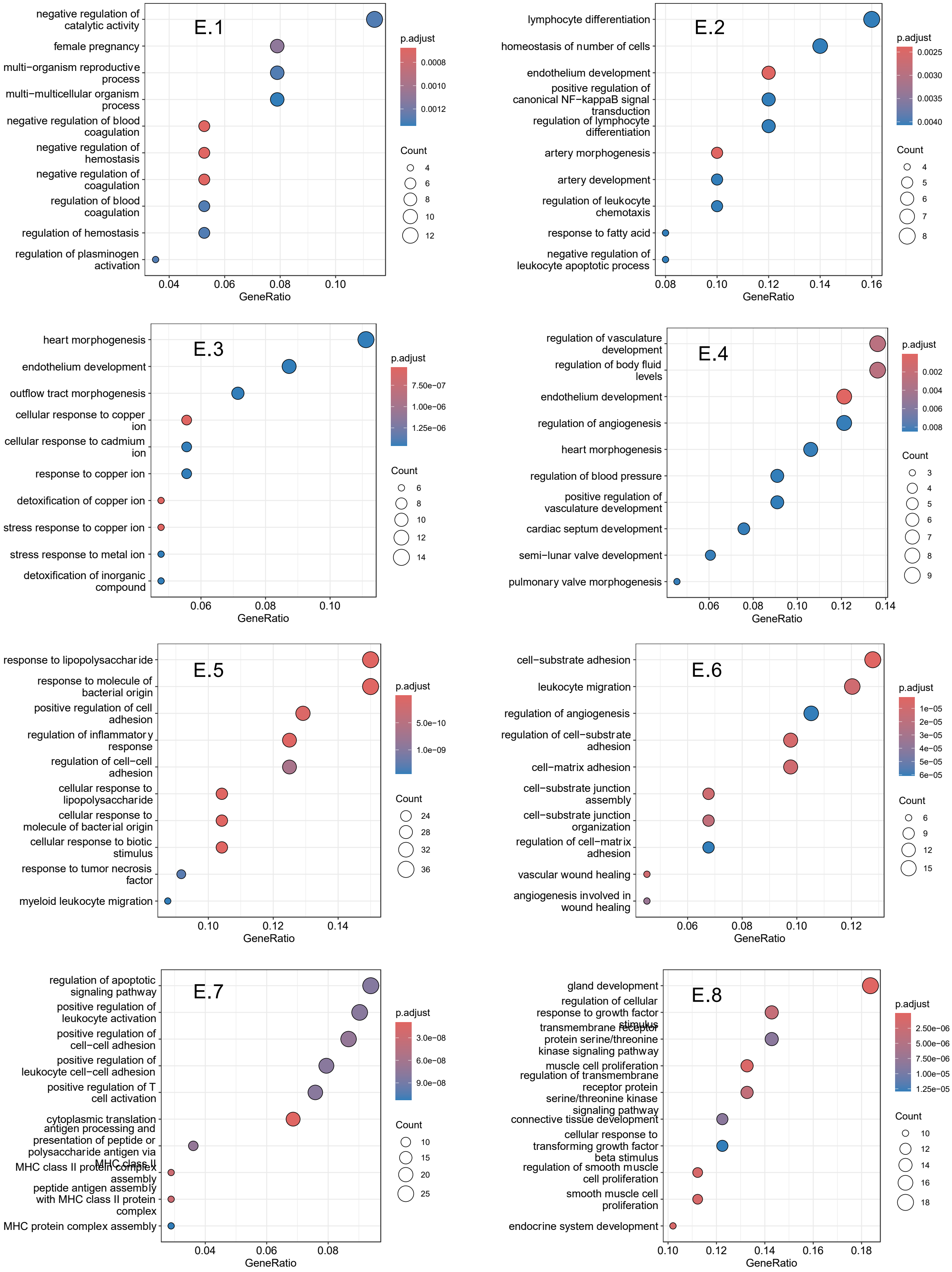

E.1
negative regulation of catalytic activity
female pregnancy
multi−organism reproductive process
multi−multicellular organism process
negative regulation of blood coagulation
negative regulation of hemostasis
negative regulation of coagulation
regulation of blood coagulation
regulation of hemostasis
regulation of plasminogen activation
0.04 0.06 0.08 0.10
GeneRatio
p.adjust
0.0008 0.0010 0.0012
Count
4 6 8 10 12
E.2
lymphocyte differentiation
homeostasis of number of cells
endothelium development
positive regulation of canonical NF−kappaB signal transduction
regulation of lymphocyte differentiation
artery morphogenesis
artery development
regulation of leukocyte chemotaxis
response to fatty acid
negative regulation of leukocyte apoptotic process
0.08 0.10 0.12 0.14 0.16
GeneRatio
p.adjust
0.0025 0.0030 0.0035 0.0040
Count
4 5 6 7 8
E.3
heart morphogenesis
endothelium development
outflow tract morphogenesis
cellular response to copper ion
cellular response to cadmium ion
response to copper ion
detoxification of copper ion
stress response to copper ion
stress response to metal ion
detoxification of inorganic compound
0.06 0.08 0.10
GeneRatio
p.adjust
7.50e−07 1.00e−06 1.25e−06
Count
6 8 10 12 14
E.4
regulation of vasculature development
regulation of body fluid levels
endothelium development
regulation of angiogenesis
heart morphogenesis
regulation of blood pressure
positive regulation of vasculature development
cardiac septum development
semi−lunar valve development
pulmonary valve morphogenesis
0.06 0.08 0.10 0.12 0.14
GeneRatio
p.adjust
0.002 0.004 0.006 0.008
Count
3 4 5 6 7 8 9
E.5
response to lipopolysaccharide
response to molecule of bacterial origin
positive regulation of cell adhesion
regulation of inflammatory response
regulation of cell−cell adhesion
cellular response to lipopolysaccharide
cellular response to molecule of bacterial origin
cellular response to biotic stimulus
response to tumor necrosis factor
myeloid leukocyte migration
0.10 0.12 0.14
GeneRatio
p.adjust
5.0e−10 1.0e−09
Count
24 28 32 36
E.6
cell−substrate adhesion
leukocyte migration
regulation of angiogenesis
regulation of cell−substrate adhesion
cell−matrix adhesion
cell−substrate junction assembly
cell−substrate junction organization
regulation of cell−matrix adhesion
vascular wound healing
angiogenesis involved in wound healing
0.06 0.08 0.10 0.12
GeneRatio
p.adjust
1e−05 2e−05 3e−05 4e−05 5e−05 6e−05
Count
6 9 12 15
E.7
regulation of apoptotic signaling pathway
positive regulation of leukocyte activation
positive regulation of cell−cell adhesion
positive regulation of leukocyte cell−cell adhesion
positive regulation of T cell activation
cytoplasmic translation
antigen processing and presentation of peptide or polysaccharide antigen via MHC class II
MHC class II protein complex assembly
peptide antigen assembly with MHC class II protein complex
MHC protein complex assembly
0.04 0.06 0.08
GeneRatio
p.adjust
3.0e−08 6.0e−08 9.0e−08
Count
10 15 20 25
E.8
gland development
regulation of cellular response to growth factor stimulus
transmembrane receptor protein serine/threonine kinase signaling pathway
muscle cell proliferation
regulation of transmembrane receptor protein serine/threonine kinase signaling pathway
connective tissue development
cellular response to transforming growth factor beta stimulus
regulation of smooth muscle cell proliferation
smooth muscle cell proliferation
endocrine system development
0.10 0.12 0.14 0.16 0.18
GeneRatio
p.adjust
2.50e−06 5.00e−06 7.50e−06 1.00e−05 1.25e−05
Count
10 12 14 16 18

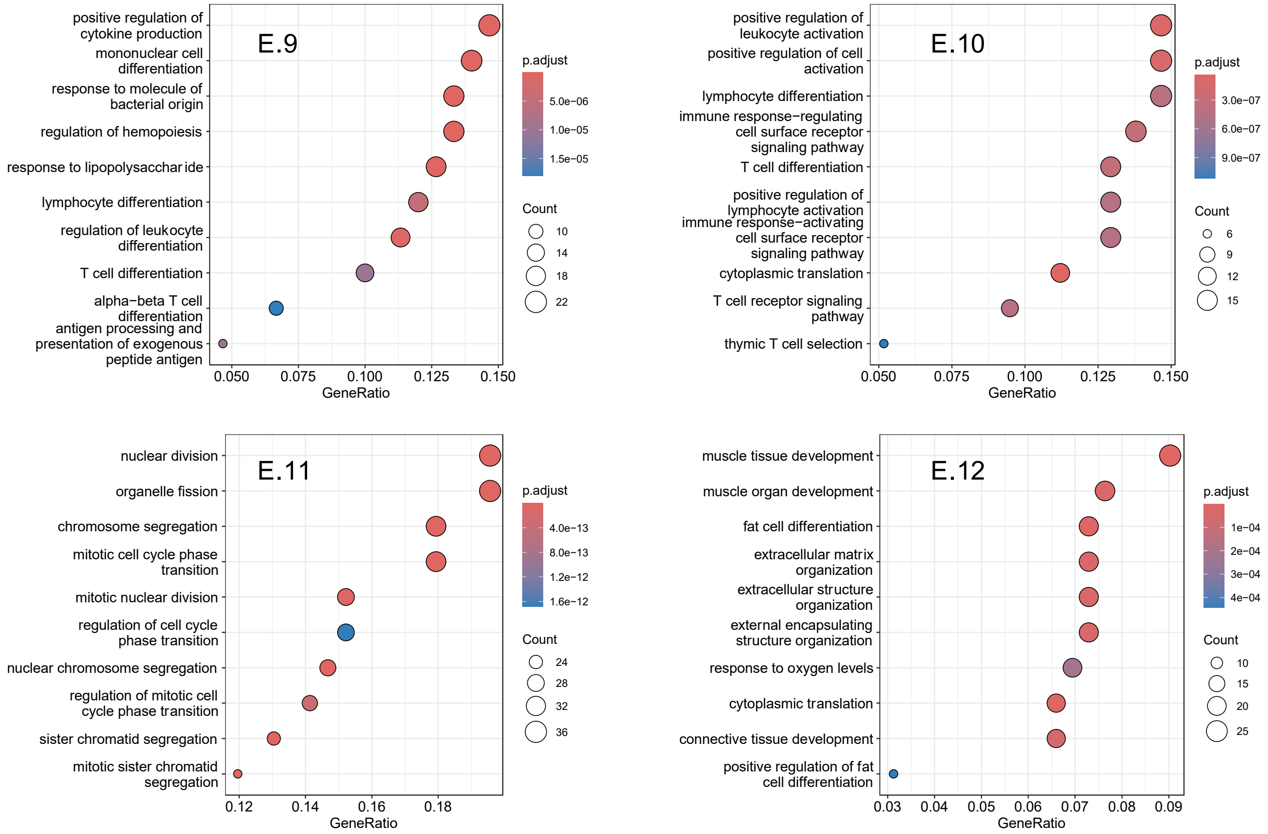

Supplementary Figure 8. GO analysis by cluster.

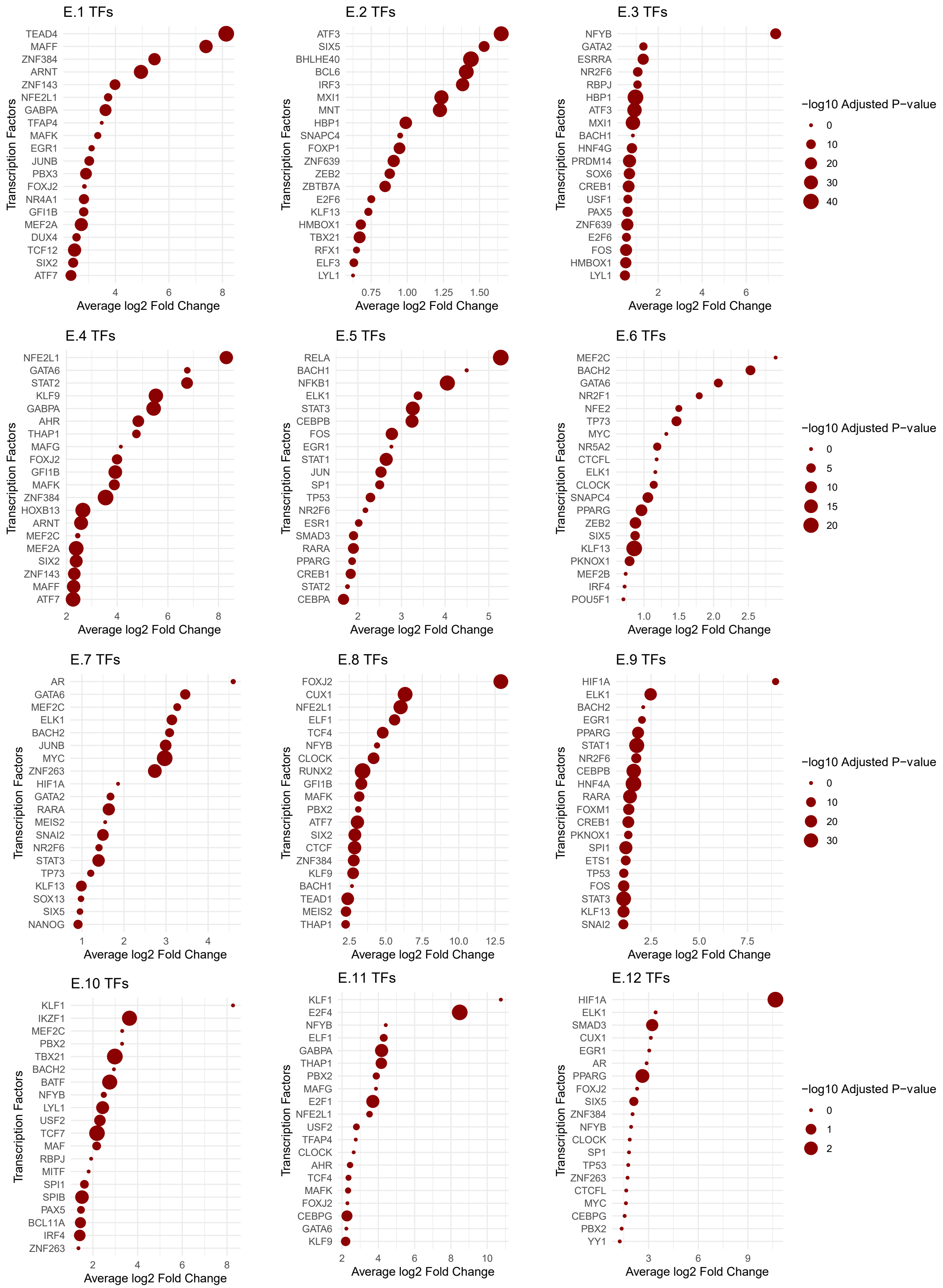

Supplementary Figure 9. Transcription Factor analysis by cluster using DoRothEA (top 20 shown).

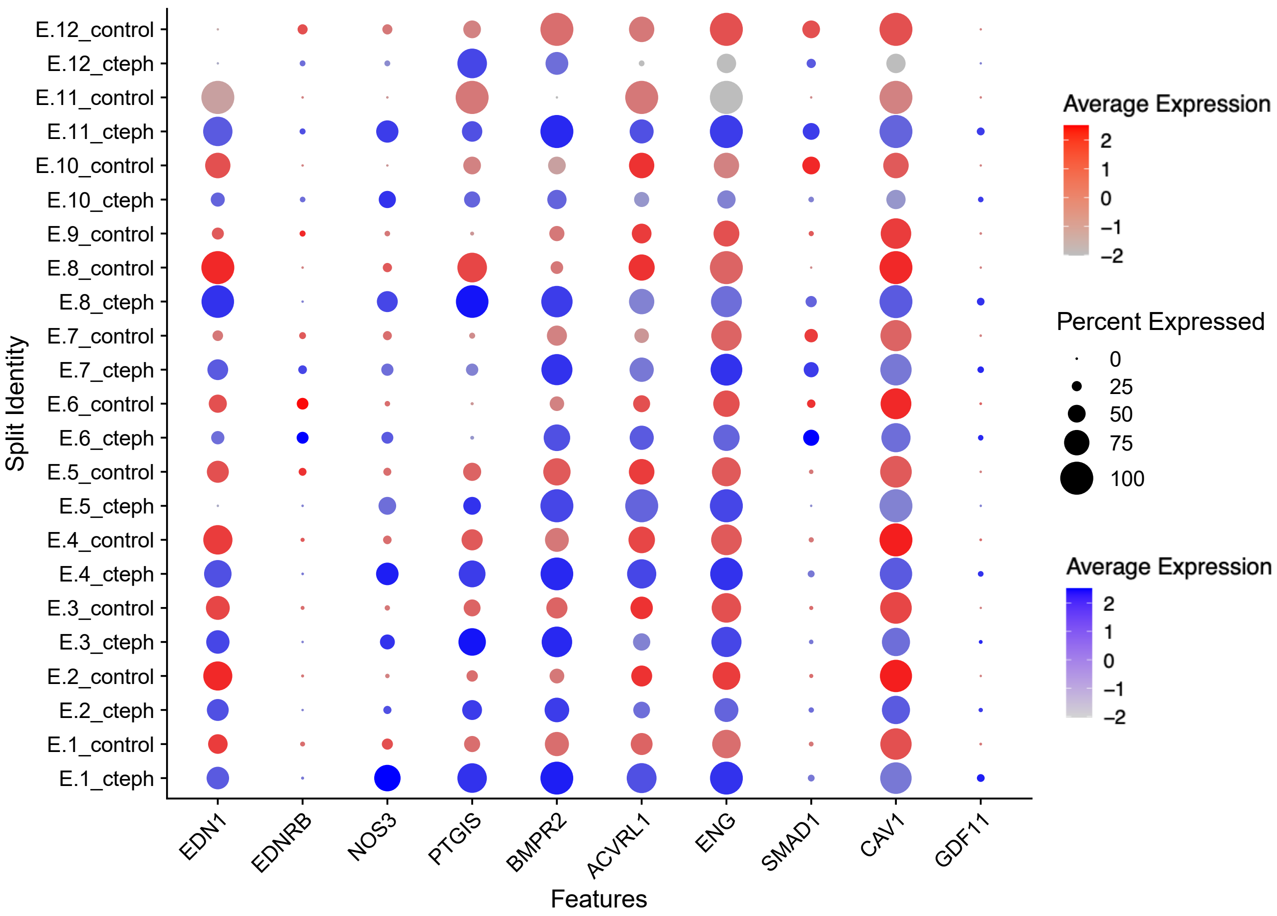

Supplementary Figure 10. Expression of selected genes within pathways targeted by current treatments for pulmonary arterial hypertension in ECs.

Need to change title to frequency

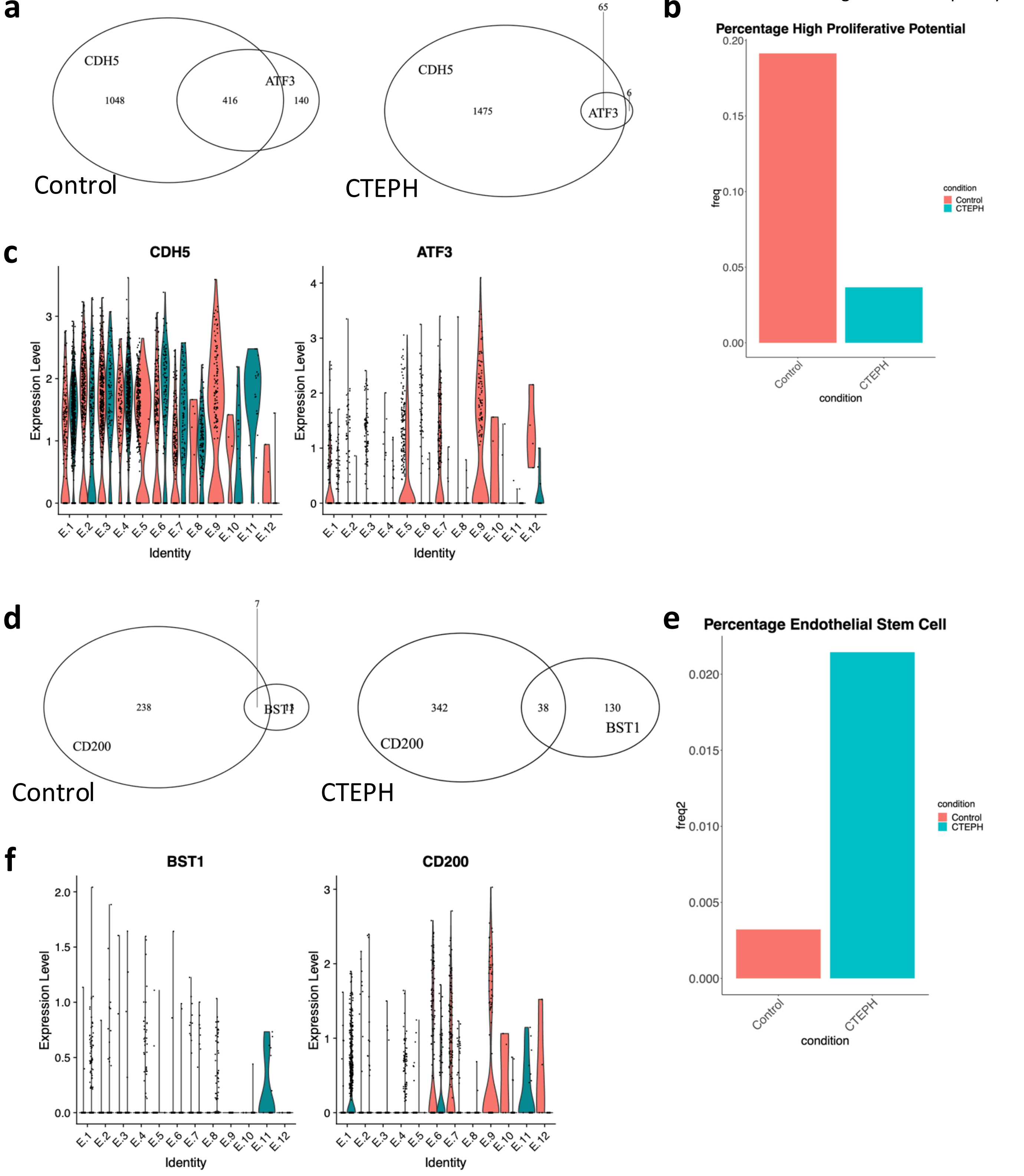

Supplementary Figure 11. Vascular repair potential in control and CTEPH endothelial cells. **a)** Venn diagrams showing expression of markers of high proliferative potential (HPP) in individual endothelial cells. Cells expressing both CDH5 and ATF3 are considered HPP. **b)** Frequency of cells expressing HPP markers. **c)** Expression of HPP markers across clusters in control (red) and CTEPH (green). **d)** Venn diagrams showing expression of endothelial stem cell markers in individual endothelial cells. **e)** Frequency of cells expressing endothelial stem cell markers. **f)** Expression of endothelial stem cell markers across clusters.

Cluster	Predominant Condition	Phenotype
**E.1**	CTEPH	Double-positive (mixed arterial/bronchial)
**E.2**	Control	Artery
**E.3**	Control	Artery
**E.4**	CTEPH	Double-positive (mixed arterial/bronchial)
**E.5**	Control	Artery
**E.6**	Control	Bronchial
**E.7**	Control	Bronchial/vein
**E.8**	CTEPH	Double-positive (mixed arterial/bronchial)
**E.9**	Control	Lymphatic
**E.10**	CTEPH	Mixed Capillary
**E.11**	CTEPH	Double-positive (mixed arterial/bronchial)
**E.12**	CTEPH	Bronchial

Supplementary Table 4. Endothelial cell clustering in control and CTEPH. Some clusters are populated predominantly by either control or CTEPH. Clusters can express markers of pulmonary artery endothelial cells, bronchial artery endothelial cells or mixed markers of multiple endothelial cell origins.

a

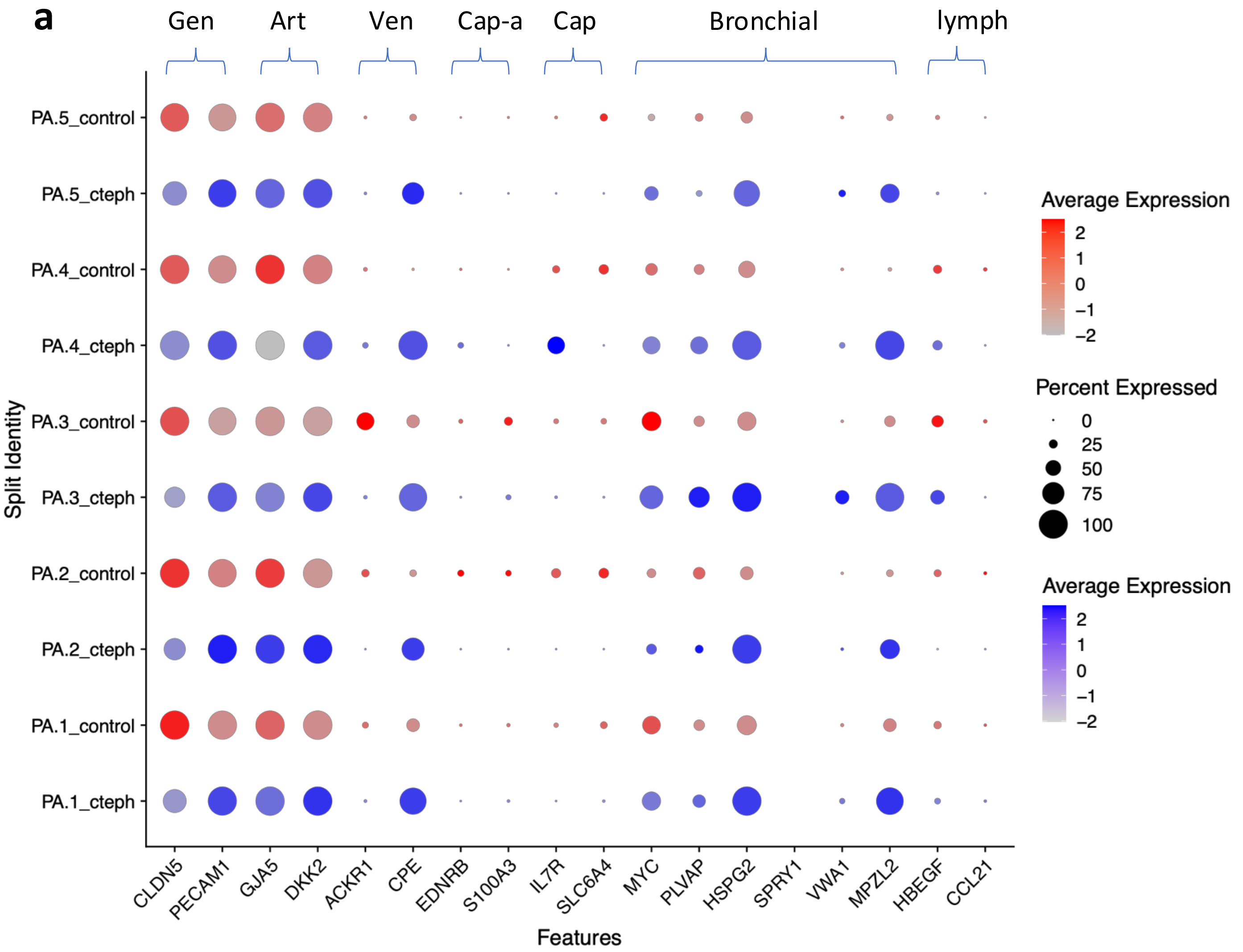

b

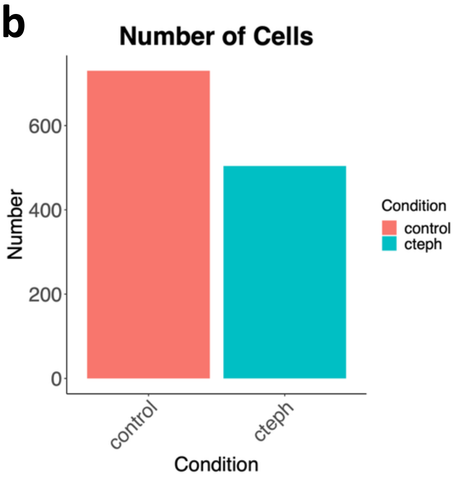

c

	Single negative (SPRY1-)	Double negative (SPRY1-, MYC-)	Triple negative (SPRY1-, MYC-, HSPG2-)
Control	730	440	254
CTEPH	504	187	3

Supplementary Figure 12. Characteristics of endothelial cells expressing pulmonary artery endothelial markers. **a)** Expression of pulmonary endothelial markers defined by the Lung Cell Atlas. Gen, general endothelial cell marker; Art, pulmonary artery; Vein, pulmonary vein; Cap-a, capillary aerocyte; Cap, general capillary cell; Bronchial, bronchial vessel; lymph, pulmonary lymphatic endothelial cell. **b)** Number of endothelial cells in each condition expressing pulmonary artery endothelial markers. **c)** Number of endothelial cells in each condition expressing pulmonary endothelial markers with negative selection for expression of bronchial artery endothelial cell markers.

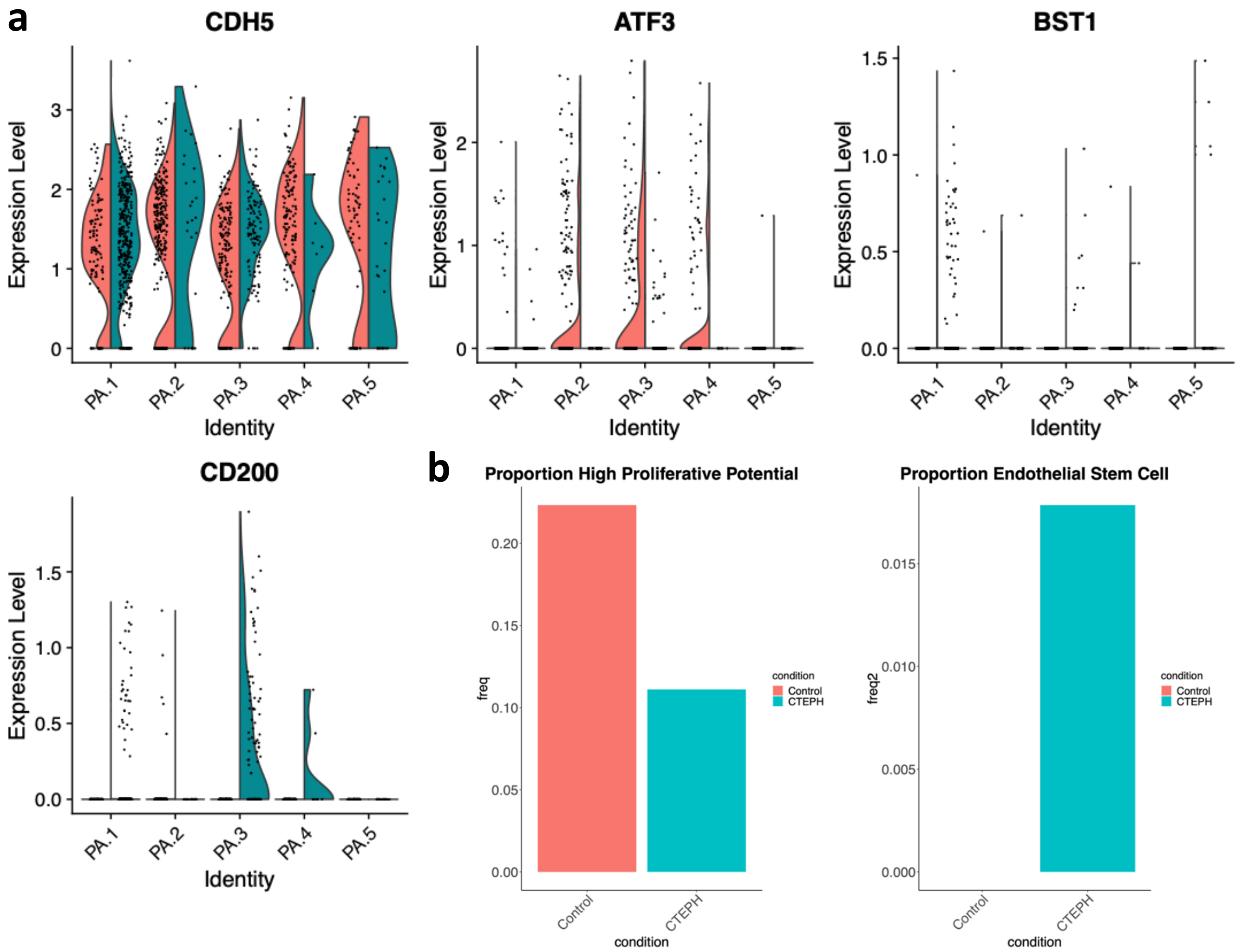

Supplementary Figure 13. Endothelial cells with properties of vascular repair in control and CTEPH pulmonary artery endothelial cells. **a)** Expression of markers of endothelial cells with high proliferative potential (CDH5, ATF3) and endothelial stem cell markers (BST1, CD200) in pulmonary artery endothelial cells. **b)** Proportion of cells with high proliferative potential (left) and of tissue-resident endothelial stem cells in pulmonary artery endothelial cells.

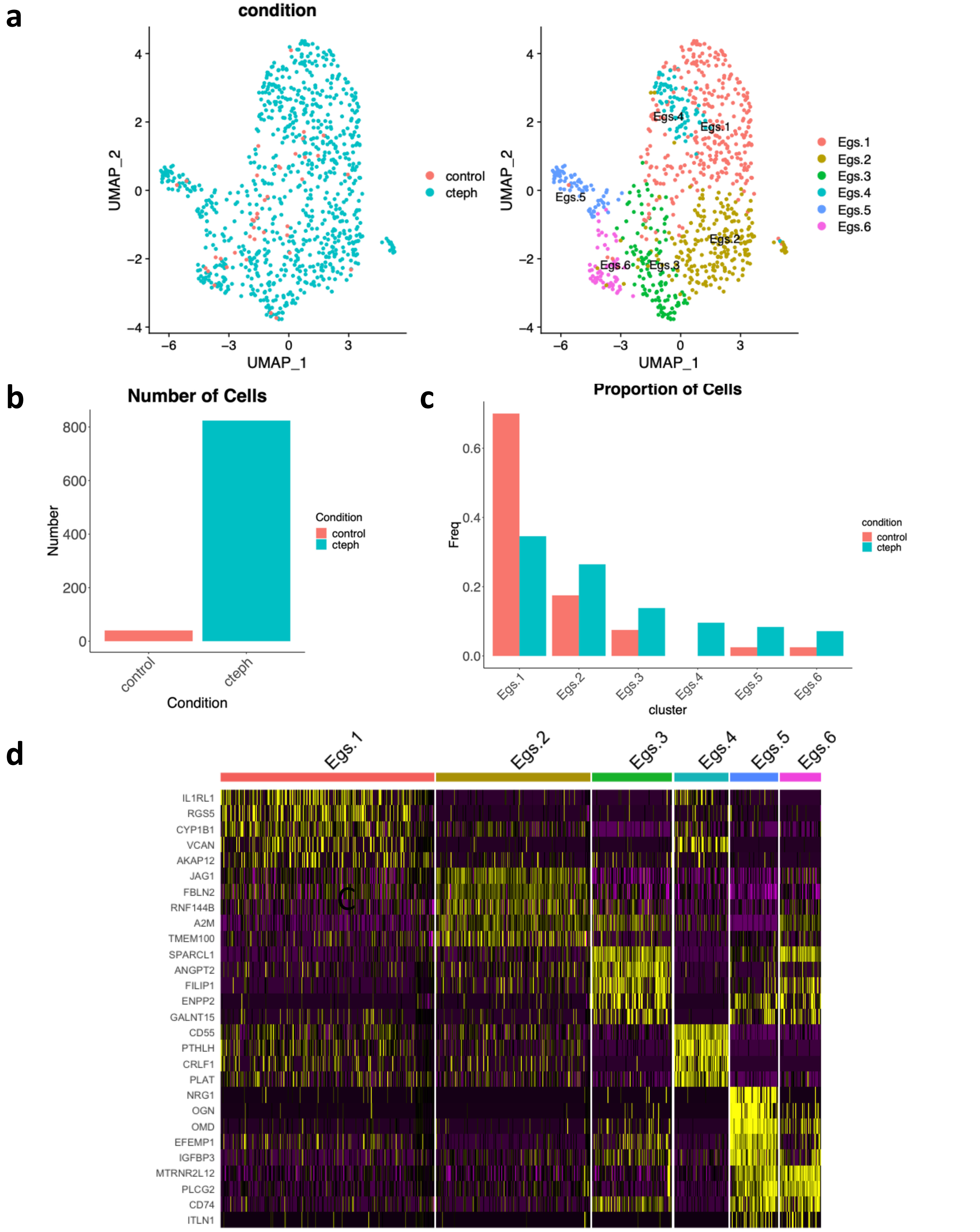

Supplementary Figure 14. Sub-clustering of endothelial cells expressing “mixed markers” (Egs) of pulmonary artery and bronchial artery endothelial cells. **a)** UMAP visualization and clustering of endothelial cells expressing “mixed markers” by condition (left) and clusters (right). **b)** Number and **c)** proportion of endothelial cells expressing “mixed markers” in each condition. **d)** Heatmap of top 5 genes per cluster of endothelial cells expressing “mixed markers”.

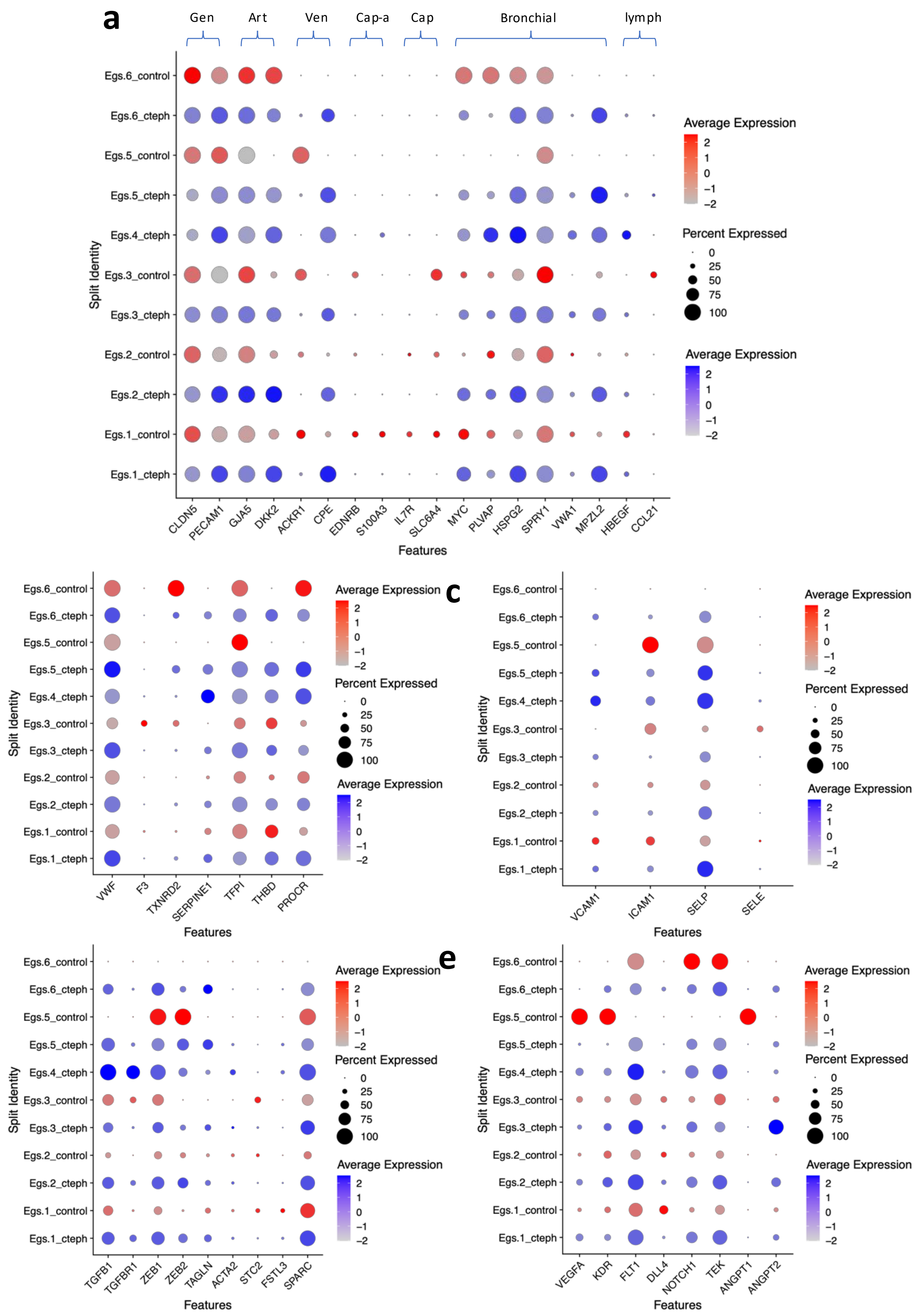

Supplementary Figure 15. Gene expression analysis of endothelial cells "double-positive" for pulmonary artery and bronchial artery EC markers. Dot plots showing gene expression of **a)** pulmonary endothelial markers, **b)** endothelial thrombotic regulation, **c)** endothelial activation, **d)** TGFβ signalling, and **e)** angiogenesis. Control ECs shown in red and CTEPH ECs shown in blue.

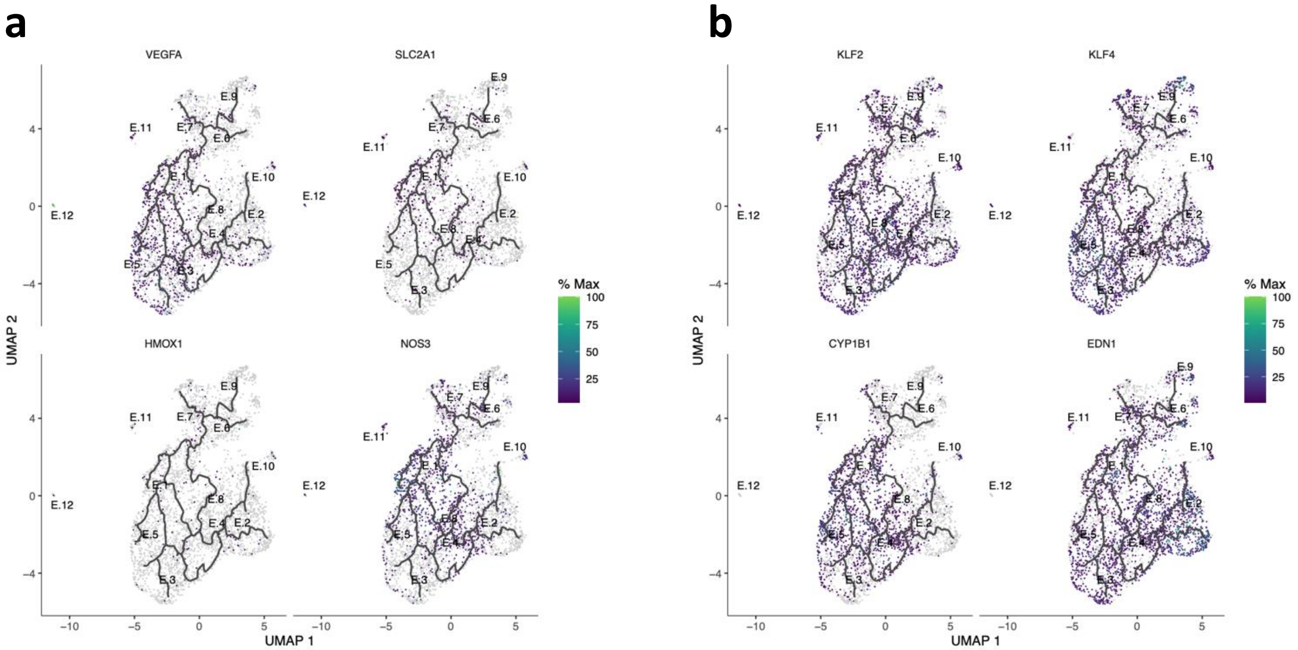

Supplementary Figure 16. Gene expression overlaid on trajectory analysis. UMAP visualization with overlaid trajectories of **a)** genes regulated with hypoxia and **b)** genes regulated by shear stress.

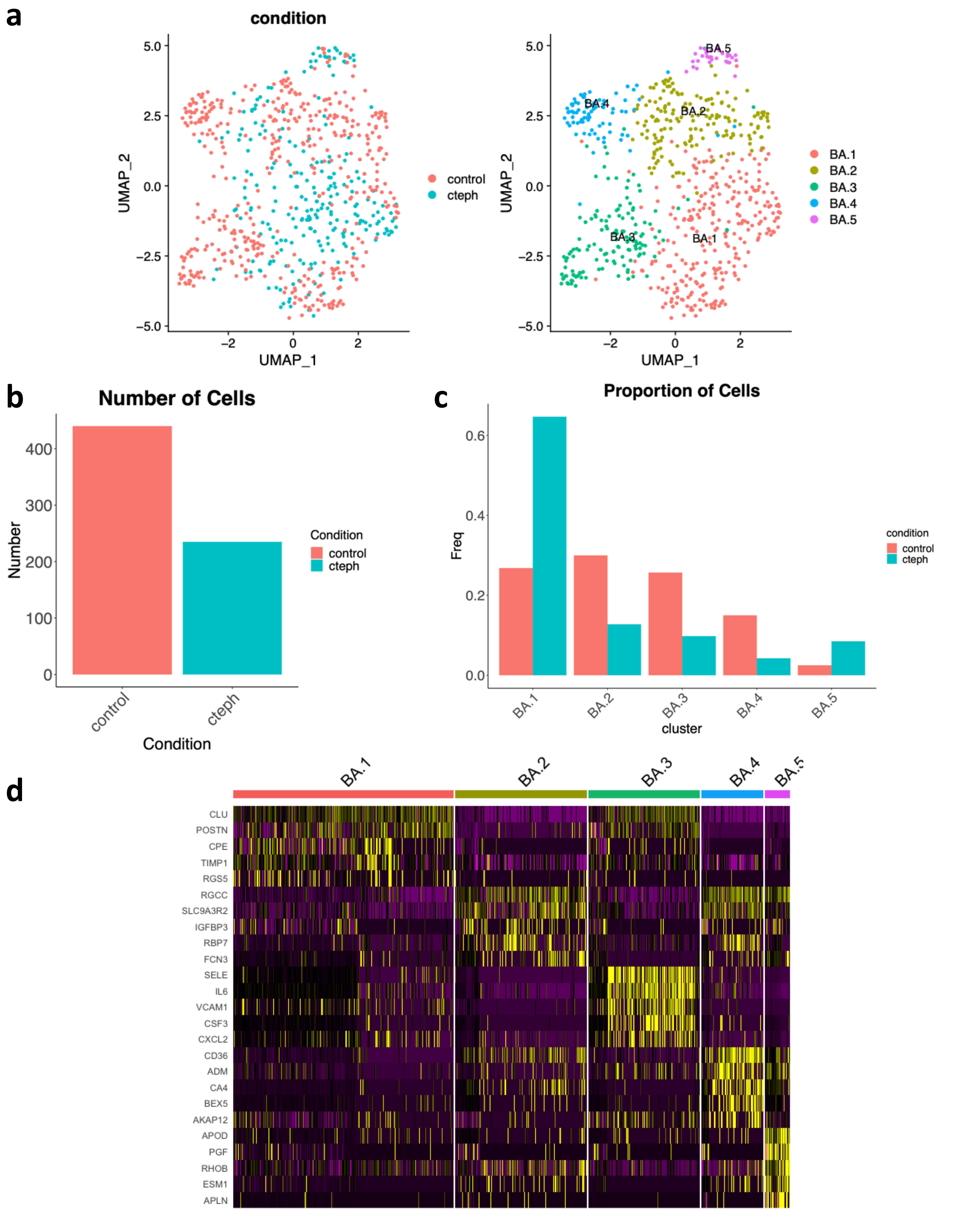

Supplementary Figure 17. Sub-clustering of bronchial artery (BA) endothelial cells. **a)** UMAP visualization and clustering of bronchial artery endothelial cells by condition (left) and clusters (right). Five bronchial artery endothelial cell clusters are noted. **b)** Number and **c)** proportion of bronchial artery endothelial cells in each condition. **d)** Heatmap of top 5 genes per cluster of bronchial artery endothelial cells.

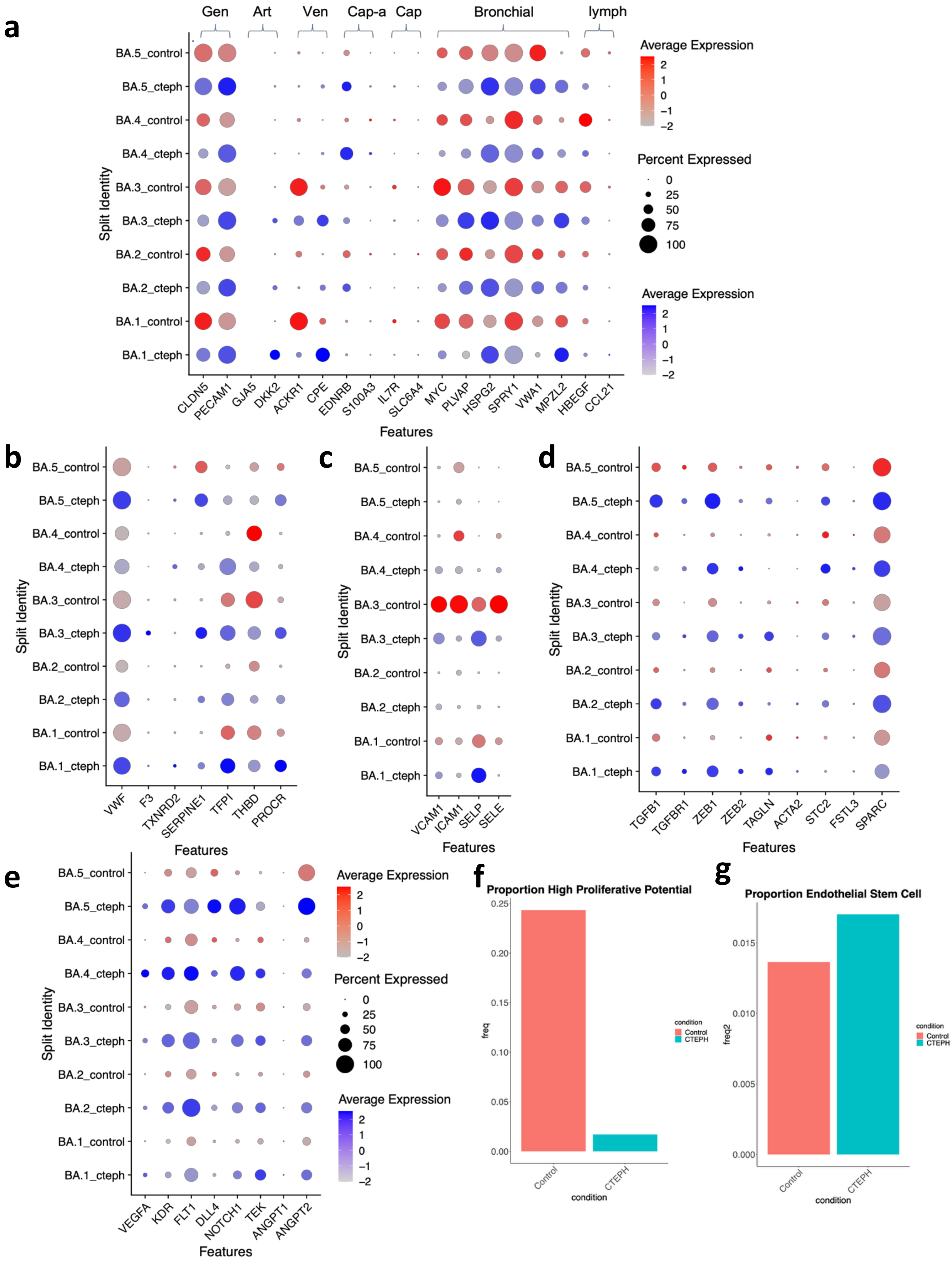

Supplementary Figure 18. Analysis of bronchial artery (BA) endothelial cells. Dot plots showing gene expression of **a)** pulmonary endothelial markers, **b)** endothelial thrombotic regulation, **c)** endothelial activation, **d)** TGFβ signalling, and **e)** angiogenesis. Control ECs shown in red and CTEPH ECs shown in blue. Proportion of **f)** high proliferative potential and **g)** endothelial stem cells.

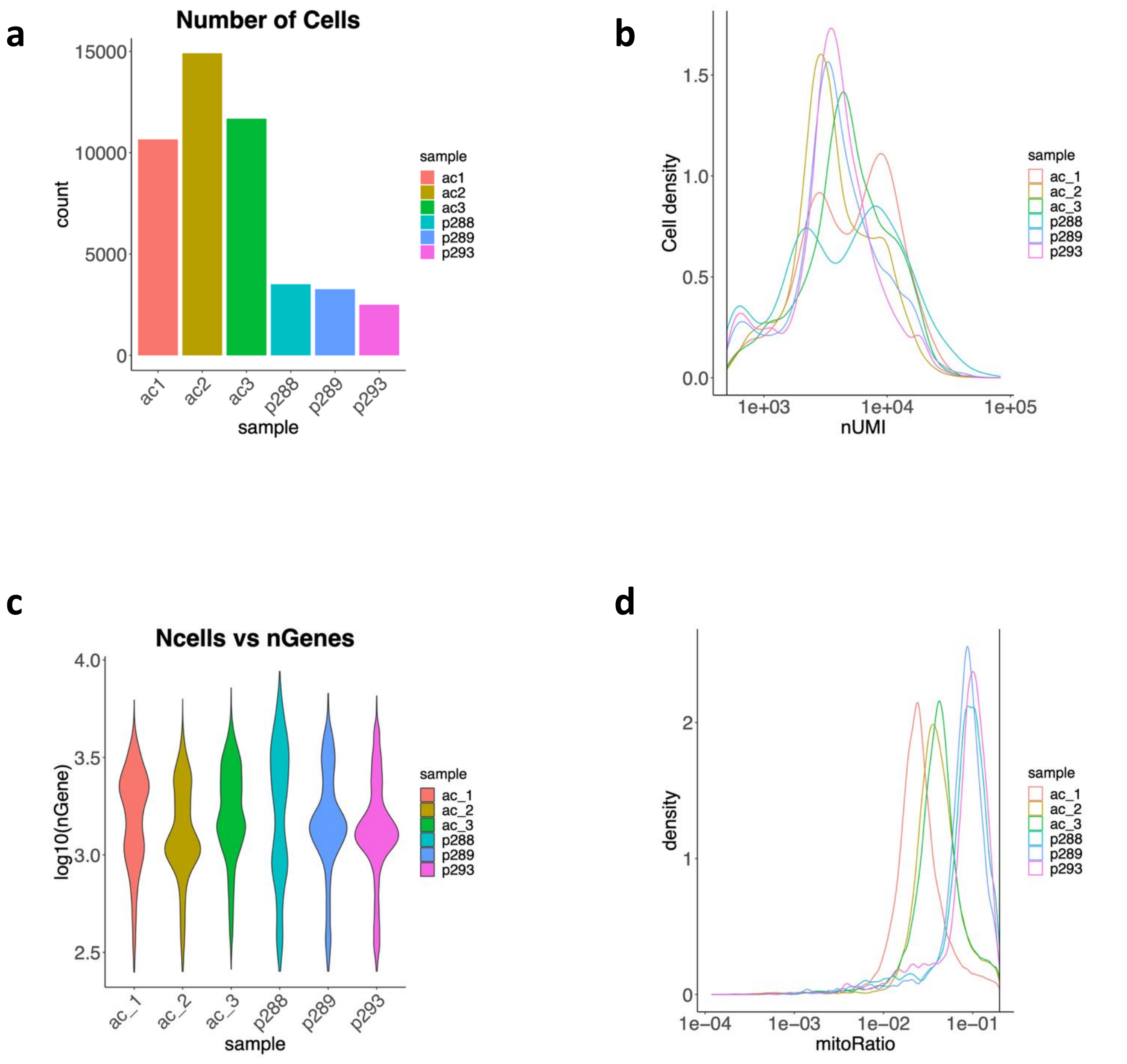

Supplementary Figure 19. Characteristics of CTEPH and atherosclerosis specimens. **a)** Number of cells in each sample for atherosclerosis (ac) and CTEPH (p). **b)** Ridge plot showing number of unique molecular identifiers (nUMI) per sample for atherosclerosis (ac) and CTEPH. **c)** Violin plot showing number of genes per sample for atherosclerosis (ac) and CTEPH. **d)** Ridge plot showing ratio of mitochondrial genes (mitoRatio) per sample for atherosclerosis (ac) and CTEPH.

a

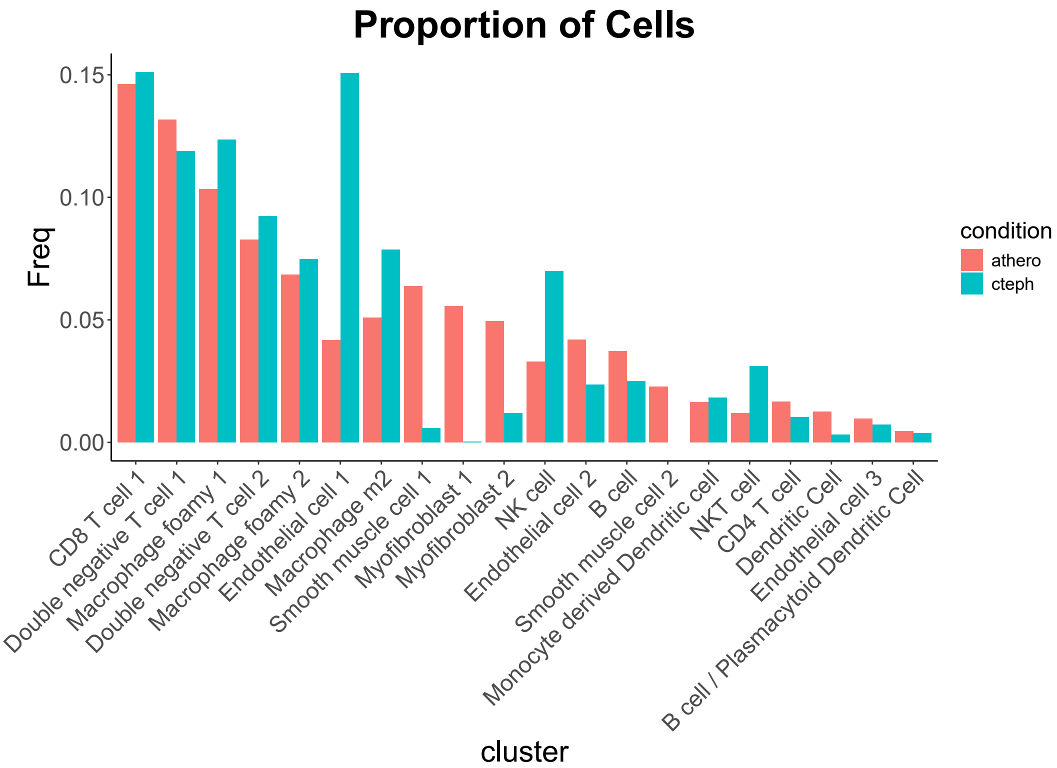

b

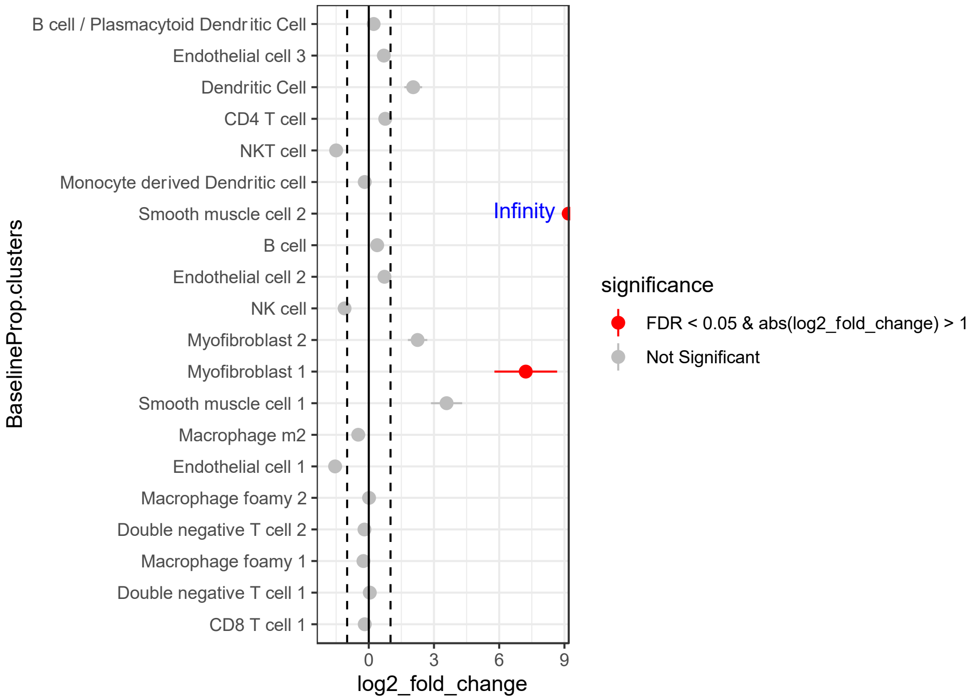

Supplementary Figure 20. Cell type Proportions. **a)** Proportion of cells for each endothelial cell cluster in control and CTEPH. **b)** Statistical analysis of cell proportion using *propeller*. Positive log2_fold_change indicates higher proportion of cells in athero and negative log2_fold_change indicates higher proportion of cells in cteph. To account for heteroskedastic variance, propeller implements two transformations to stabilize variance prior to testing statistical differences using a linear modelling framework. Therefore, propeller does not report variance and the displayed error bars represent 20% of log2_fold_change. In this visualization, a red colour indicates statistical significance.

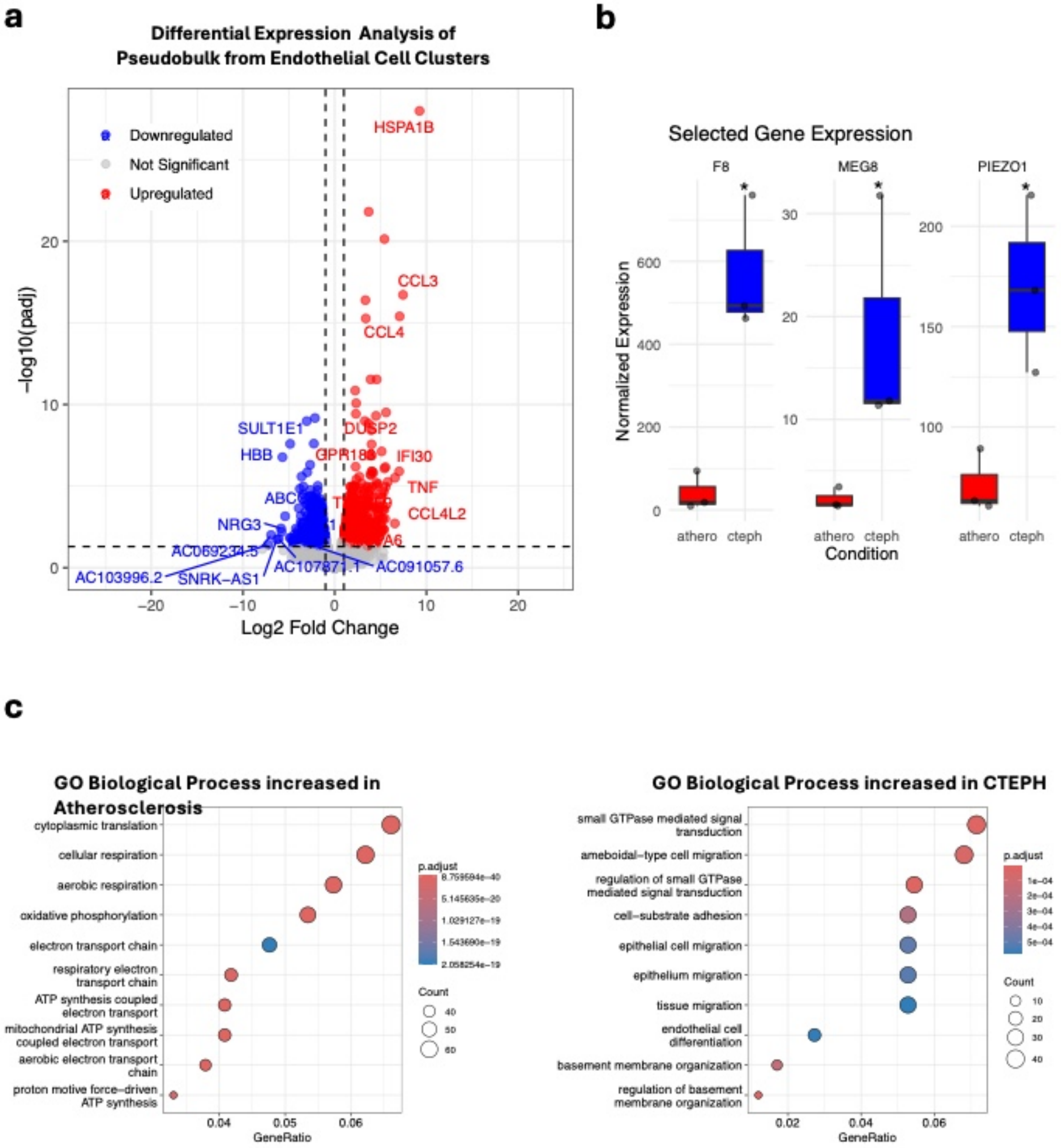

Supplementary Figure 21. Pseudobulk analysis of subclustered endothelial cells. **a)** Volcano plot of differentially expressed genes. Upregulated genes (red) represent genes upregulated in atherosclerosis. Statistically significant downregulated genes (blue) represent genes upregulated in CTEPH. Analysis was performed using DESeq2. Statistical significance defined by fold change > 2 and Benjamini-Hochberg adjusted p-value < 0.05. **b)** Boxplots showing expression of selected genes upregulated in CTEPH. * indicates adjusted p-value < 0.05. c) GO “biological process” analysis of GO biological process terms upregulated in atherosclerosis (left) and CTEPH (right).

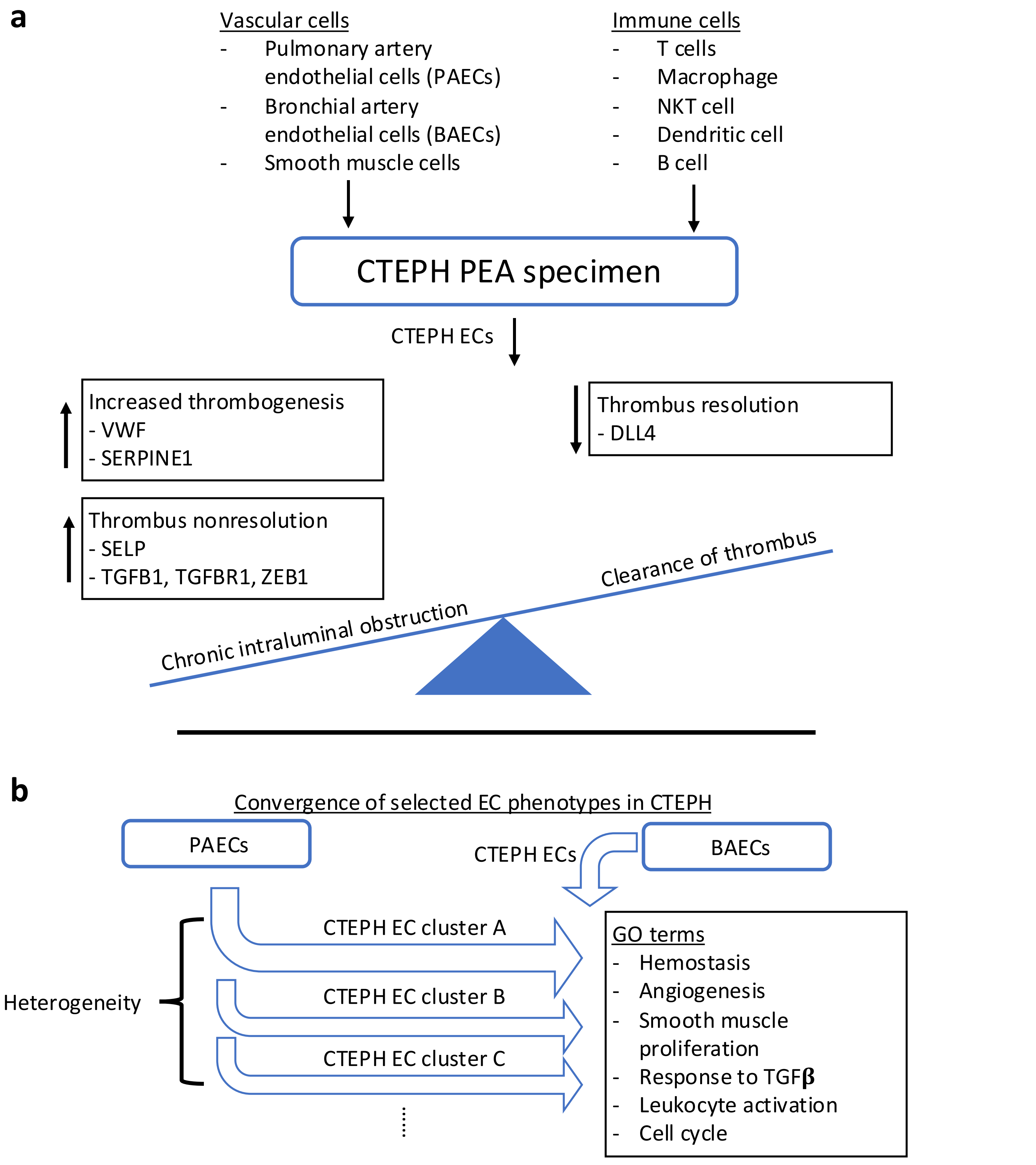

Supplementary Figure 22. Schematic of endothelial pathology in CTEPH. a) CTEPH is a vascular inflammatory disorder with a complex complement of cell types. CTEPH ECs display patterns of disrupted homeostasis of thrombogenesis and thrombus resolution that may contribute to chronic intraluminal obstruction and fibrous organization of thrombus. b) CTEPH ECs often co-express markers of both PAECs and BAECs. There is significant heterogeneity of ECs with multiple clusters that diverge from control PAECs and BAECs. GO terms for clusters with greater numbers of CTEPH ECs compared to control are shown.
